# MultipleXLab: A high-throughput portable live-imaging root phenotyping platform using deep learning and computer vision

**DOI:** 10.1186/s13007-022-00864-4

**Published:** 2022-03-27

**Authors:** Vinicius Lube, Mehmet Alican Noyan, Alexander Przybysz, Khaled Salama, Ikram Blilou

**Affiliations:** 1 Laboratory of Plant Cell and Developmental Biology (LPCDB), Biological and Environmental Sciences and Engineering (BESE), King Abdullah University of Science and Technology (KAUST), Thuwal, 23955-6900 Saudi Arabia; 2 Ipsumio B.V., High Tech Campus, 5656 Eindhoven, AE Netherlands; 3 Sensors Lab, Advanced Membranes and Porous Materials Center (AMPMC), Computer, Electrical and Mathematical Science and Engineering (CEMSE), KAUST, Thuwal, Saudi Arabia

**Keywords:** Phenomics, Machine learning, Image segmentation, Automation, CNC microscope

## Abstract

**Background:**

Profiling the plant root architecture is vital for selecting resilient crops that can efficiently take up water and nutrients. The high-performance imaging tools available to study root-growth dynamics with the optimal resolution are costly and stationary. In addition, performing nondestructive high-throughput phenotyping to extract the structural and morphological features of roots remains challenging.

**Results:**

We developed the Multiple**XL**ab: a modular, mobile, and cost-effective setup to tackle these limitations. The system can continuously monitor thousands of seeds from germination to root development based on a conventional camera attached to a motorized multiaxis-rotational stage and custom-built 3D-printed plate holder with integrated light-emitting diode lighting. We also developed an image segmentation model based on deep learning that allows the users to analyze the data automatically. We tested the Multiple**XL**ab to monitor seed germination and root growth of Arabidopsis developmental, cell cycle, and auxin transport mutants non-invasively at high-throughput and showed that the system provides robust data and allows precise evaluation of germination index and hourly growth rate between mutants.

**Conclusion:**

Multiple**XL**ab provides a flexible and user-friendly root phenotyping platform that is an attractive mobile alternative to high-end imaging platforms and stationary growth chambers. It can be used in numerous applications by plant biologists, the seed industry, crop scientists, and breeding companies.

**Supplementary Information:**

The online version contains supplementary material available at 10.1186/s13007-022-00864-4.

## Background

With the global increase in food demand, selecting crops that perform well is critical to improving food production. Breeding for better crops includes selecting desirable phenotypic traits. However, isolating plants with particular phenotypes accurately and nondestructively is still challenging because it involves analyzing hundreds to thousands of samples and sometimes selecting specific complex features at multiscale levels ranging from the cell, tissue, and organ to the whole plant [1, 2]. Many platforms are available to achieve multimodal and multidimensional phenotyping. These platforms can operate in a wide range of conditions ranging from controlled and semi-controlled environments to field conditions [2, 3]. High-throughput phenotyping is performed primarily at the organism and organ levels and has a limited resolution. Examples of these platforms include *PlantScreen*^*TM*^* Systems*, equipped with a three-dimensional (3D) laser and multispectral camera and have a range of instruments that allow phenotyping in growth chambers, greenhouses, and the field.^1^ Some systems can also phenotype the roots grown in either Rhizotrons^2^ [4] or *Rhizotubes*^3^ [5]. The *PlantEye* can automatically image and compute multiple above-ground features nondestructively^4^ [6]. Other platforms are tailored to more specific traits, such as *Phenopsis*, which monitors the plant response to a water deficit [7]. In addition, *LiDAR* is a creative system for high-throughput phenotyping in the field [8, 9]. Additional platforms have also been valuable for live imaging. The 3D root growth and imaging system allows high-throughput phenotyping of rice root traits at the seedling stage [10]. *RhizoChamber* is a robotic platform used to analyze root growth in rhizoboxes [11]. The system integrates hardware and software to analyze the spatio-temporal dynamics of root growth from time-course images of multiple plants. Although these systems allow high-throughput phenotyping, they are costly. Another attractive system to monitor root growth noninvasively is X-ray Computed Tomography, which allows 3D root phenotyping in soil [12]. However, the system does not allow high-throughput phenotyping, the resolution is low and is also costly. Recently, efforts have been put forward to establish alternative approaches for low-cost live imaging of plants under stress conditions [13, 14]. For higher magnification imaging, the most commonly used systems are costly stereomicroscopes coupled with digital cameras. The proposed hybrid mini-microscope offers an alternative low-cost tool that combines physical and optical magnification to achieve high magnification and multifluorescence imaging [15, 16]. Although some of these platforms are easy to operate, and images can be captured rapidly, they are often heavy and nonportable, and the setup is inflexible. Moreover, they allow only one mode of imaging. High-throughput phenotyping often comes at the expense of resolution, whereas phenotyping at a high spatial and temporal resolution is difficult to achieve at large scales.

This study proposes a platform that combines high-throughput phenotyping with high resolution. The platform can be used as a single-plate system to monitor biological processes at a high resolution. Multiple**XL**ab is an optical, modular imaging setup for high-throughput phenotyping of seed germination and early root growth on agar and soil plates. Multiple**XL**ab is based on off-the-shelf, low-cost, portable camera components that we modified and adapted to capture dynamic processes noninvasively in living biological systems. The system comprises a digital camera and two different types of 3D-printed multiplate holders with integrated growth LED lighting. Users can simultaneously capture 18 square Petri dish plates containing multiple specimens, allowing screening of up to thousands of Arabidopsis seedlings to monitor germination rates and root-growth dynamics noninvasively. The system can acquire and analyze up to 100 images per hour, with each image having up to 64 seeds or roots, which allows automated imaging of thousands of Arabidopsis and hundreds of tomato seedlings growing on agar plates at a high resolution. We also implemented computer-vision and pattern recognition technologies combined with machine learning to analyze and quantify distinct phenotypes. We used Multiple**XL**ab to determine differences in seed germination index and growth rates of developmental, auxin, and cell-cycle mutants.

We demonstrate that Multiple**XL**ab has exceptional resolution for imaging worms, soil nematodes, insect behavior, and feeding habits in natural habitats. The system is highly flexible and can be adapted to perform dual-axis imaging of single plates, allowing both vertical or horizontal camera/lens orientations simply by flipping the entire setup without the need to unscrew or turn a knob. Multiple**XL**ab is ideal for mutant screening, where millions of seeds must be scored for a particular phenotype.

## Results

### Building a low-cost, high-resolution imaging system

**Fig. 1:**
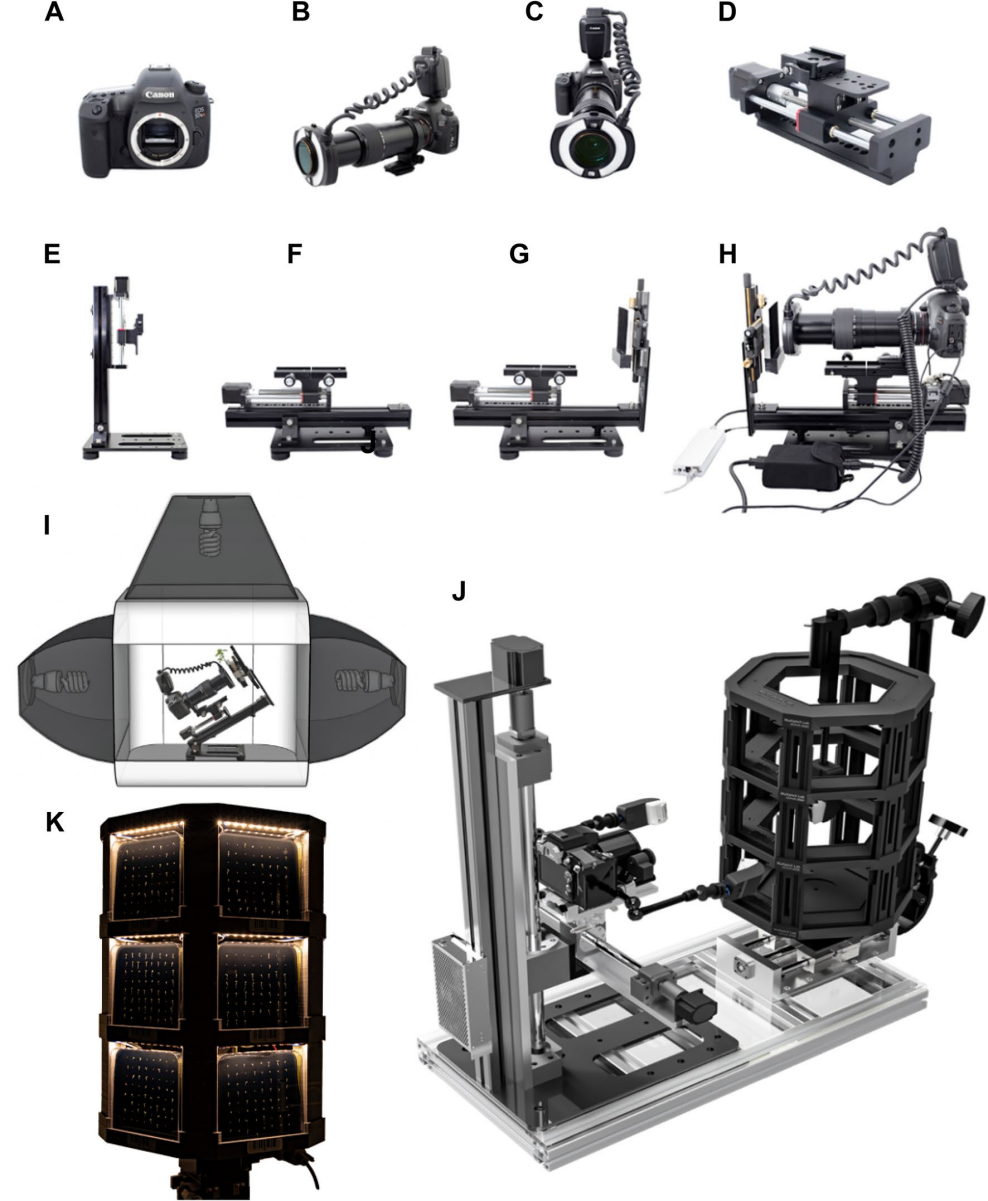
Components of the imaging setup and automated Multiple**XL**ab. **A** the camera body; **B** perspective view of the camera attached with lens and visible bandwidth pass filter, teleconverter, and ring flash unit; **C** front view of **B**; **D** step-motor module; **E** stage with z-stepper rail assembled vertically and **F**) horizontally; **G** *x–y* micropositioning sample stage with a black velvet backdrop attached to the camera stage; **H** assembled imaging setup composed of an off-the-shelf motorized stage, camera, and power components; **I** lightbox providing soft and diffuse continuous lighting. The total weight of the single-plate imaging setup is under 8 kg; **J** Multiple**XL**ab computer-generated image showcasing the prototype used to achieve high-throughput screening to capture macro-to-micro scale images of plants. Multiple**XL**ab weighs less than 25 kg. Petri dish plates mounted on a 3D-printed multiplate carousel **K**. *N* = *1152*. *N* is the number of plants used in this study

The single-plate imaging setup comprises a digital Canon 5DSr SLR camera devoid of a low-pass filter and replaced by a full spectrum filter made from fused silica. High magnification is achieved using the Canon MP-E 65 mm f/2.8 1–5× lens [17] stacked on a 2:1 teleconverter to achieve nearly 10:1 magnification. The camera system’s position is actuated with micro-steps using a vertical/horizontal motorized rail (Fig. 1A to H, Additional file 1: Table S1). This setup provides a field of view (FOV) of 36 × 24 mm at 1× magnification (life-size 1:1) and 3.88 × 2.55 mm at 9–10× (life-size 10:1) magnification using the teleconverter.

### Implementation for biological systems

**Fig. 2:**
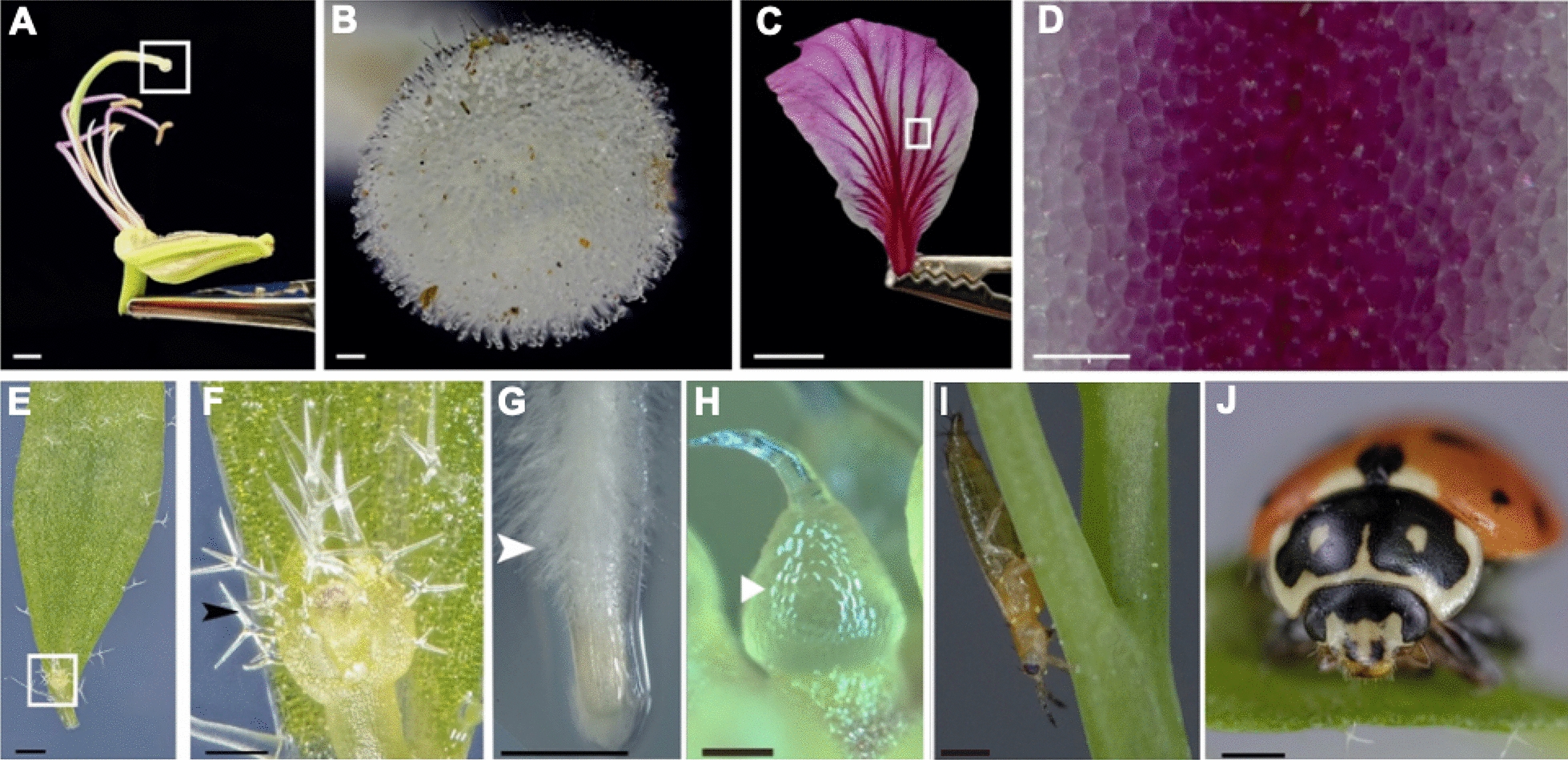
Imaging plant organs. **A** Flower of *Hibiscus rosa-Sinensis* L. (*N* = *3*); **B** enlarged view of the stigma in **A** using 61 stacked images; **C** petal from *Hibiscus rosa-Sinensis* L. (*N* = *5*); **D** enlarged view of petal in **C** using 13 stacked images; **E**) *Arabidopsis thaliana* mature leaf with the flower bud enlarged in **F**) using 20 stacked images (*N* = *5*); **G** root tip of tomato roots growing on agar (*N* = *10*); **H** handheld 5:1 magnification photograph of a juvenile cucumber fruit growing in the greenhouse (note the ring flash reflects the cellular structure, making its boundaries more pronounced) (arrow) (*N* = *20*); **I** single shot of a *Thysanoptera* in an Arabidopsis stem (*N* = *15*); **J**) image of a ladybird beetle (*Harmonia axyridis*) on Arabidopsis leaves using 40 stacked images (*N* = *1*). *N* is the number of plants used in this study. Scale bars: **A** and **C** 10.0 mm, **B** 0.04 mm, **D** 0.1 mm, **E** 2.4 mm, **F** 0.8 mm, **G** 0.25 mm, **H** and **I** 0.5 mm, and **J** 0.61 mm

To evaluate the quality of the single-plate imaging system, we first imaged organs from different plant species using Arabidopsis and wild-grown specimens (Fig. 2A–G), including flowers, leaves, and roots, which demonstrates that this setup can provide high-quality, detailed images (Fig. 2A to F). The system enabled the visualization of tissue layers and fine structures, such as the stigma papillae on a flower carpel of Hibiscus, at the micrometer scale (Fig. 2A, B), and the cell morphology and organization of epidermal layers of a Hibiscus petal flower (Fig. 2C, D), flower buds (Fig. 2E) and trichomes of Arabidopsis (Fig. 2F), and root hairs of tomato plants (Fig. 2G). Using the system handheld for 5:1 photomacrography assisted by a ring flash attached to the lens provided excellent images of trichomes on a cucumber (Fig. 2H). We were also able to detect thrips infesting Arabidopsis (Fig. 2I, Additional file 2: Movie S1) and acquired images with the detailed anatomy of a ladybird beetle (Fig. 2J), where we observed the eyes, antennae, and mouthparts, such as the maxillary palpus with sensory hairs of 100 to 200 μm in length (Fig. 2J).

### Automatizing image acquisition

We sought to automate image acquisition and facilitate stacking to increase the system potential (Fig. 1H to J). Thus, we micropositioned the camera system with the aid of stepper motors capable of reaching a maximum resolution of 1 μm per step, which facilitates refocusing and acquiring z-stacks to mitigate the downsides of a shallow depth-of-field (DOF) in extreme macrophotography [18]. This feature enables the end user to maximize the optical resolution of the imaging system by increasing the numerical aperture of the lens to its optimal maximum. This maximization results in shallow DOF images that can be automatically stacked and systematically masked using computer software and post-processing workflows, producing an overall sharper image with an extended DOF [19].

### Time-lapse live imaging

We implemented this setup to capture dynamic biological processes in challenging environments using the optical arrangement and resolution obtained above. To this end, we first optimized the time-lapse imaging of plant samples using the camera’s built-in intervalometer to establish an adequate frequency for visualizing dynamic processes. This setup allowed us to operate the camera autonomously and use a computer to control the system for more specific and intricate tasks, such as automated focus stacking operations.

Because plant roots grow underground, monitoring changes during growth noninvasively in time and space is challenging. To overcome this challenge, we used the customized mini-rhizotron system (Additional file 1: Fig. S1). We latched this mini-rhizotron onto the single-plate camera stage using the camera’s versatile orientation (horizontal in this case) and noninvasively monitored Arabidopsis roots for three days, and tomato root growth and lateral root behavior for up to 14 days (Additional file 3: Movie S2).

With this setup, we detected microscopic worms and free-living nematodes interacting with the tomato root surface in the soil (Additional file 4: Movie S3) and insects feeding off the surface of a tomato root (Additional file 5: Movie S4). Because the system is portable, we tested its long-term capabilities outside the laboratory by placing it in the greenhouse and monitoring root recovery after wounding using tomato plants (Additional file 1: Fig. S2, A to J). Our live time-lapse imaging system allowed us to determine the recovery time for roots after wounding and monitor the root regeneration process occurring after 45 to 50 hours in cut roots (Additional file 1: Fig. S2G and Additional file 6: Movie S5).

### Optical and imaging system performance

**Fig. 3:**
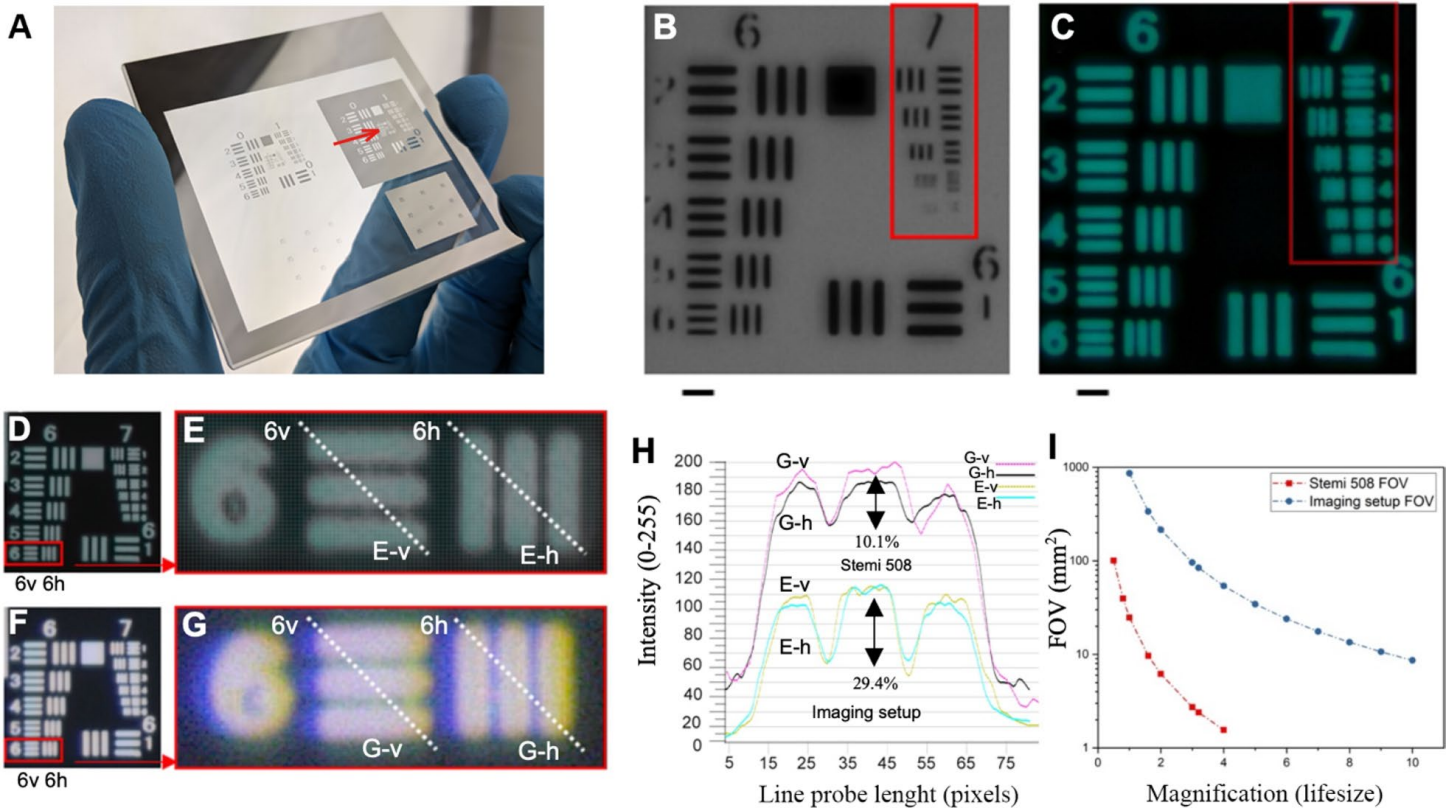
Imaging performance. **A** 1951 USAF resolution target (63 × 63 × 2 mm) on the stereo microscope stage. Red arrow highlights the group element used for optical benchmarking; **B** and **C** depict groups 6 and 7 from both positive **B** and negative **C** chrome patterns, respectively. The contrast of the modulation frequency of 114 lp/mm obtained from the resolution target was evaluated on the negative group 6—element 6 using the imaging setup and stereo microscope. The contrast tests were performed at maximum native 5:1 and 4:1 magnifications for **D** the imaging setup and **F** stereomicroscope systems, respectively; **E** and **G** are crops from **D** and **F**, respectively. The vertical and horizontal intensity line probes on element 6 are depicted in (**E**-v) and (**E**–h) and in (**G**-v) and (**G**-h) for the imaging setup and Stemi 508, respectively; (**H**) an average three-fold superior contrast between the imaging setup (**E**-v) and (**E**–h) (**29.4%**) compared to the Stemi 508 (**G**-v) and (**G**-h) (**10.1%**) is shown in the intensity gradient from horizontal and vertical line pairs. The line probe intensity values for (**G**-v) and (**G**-h) have a higher absolute value, despite the lower contrast. This attenuated brightness was challenging to control due to the intense chromatic aberration in the brightfield illumination using the Stemi 508; **I** field of view (FOV) comparison between the two imaging systems depicting the much larger FOV in the imaging setup across the entire corresponding magnification range. Scale bars: **B** and **C** 0.02 mm

To assess the optical performance of the proposed system, we compared it with the similarly priced Stemi 508 stereomicroscope. To this aim we used a resolution target also termed test chart that allows testing and comparing the optical performance of the two systems. The chart consists of multiple precision chrome patterns on a glass substrate (Fig. 3A–G). We used the standard target chart USAF 1951 that contains 7 groups and each group contains 6 elements (Fig. 3A) and each element has three lines with a constant spacing (Fig. 3D–G). We used this chart to test the ability of both systems to transfer the modulation frequency, in similar conditions to the experimental settings to compare contrast at the pixel level.

We found that, for both systems, the resolution target could be used for up to element 6 in group 6 (114 line pairs per millimeter [lp/mm]; Fig. 3D–F). Beyond element 1 of group 7, the resolution target became visibly unreliable for both positive and negative patterns in both imaging systems, as the pattern on the mask had manufacturing issues (Fig. 3B, C, red box). Hence, we used the modulation transfer function response from the imaging setup (Fig. 3E) and Stemi 508 (Fig. 3G) from element 6 in group 6 to evaluate the difference in contrast at that modulation frequency (114 lp/mm; Fig. 3H). We acquired the target image in the center of the lens/image from both systems. We performed the contrast tests at 4:1 and 5:1 magnification, the maximum native zoom of the stereomicroscope and the proposed imaging setup, respectively. From the vertical and horizontal intensity line probes assessed on element 6, we compared the contrast of 29.4% from the imaging setup (Fig. 3E-v and E-h) with the Stemi 508 (Fig. 3G-v and G-h) of 10.1%, indicating higher contrast and higher resolving power using the proposed imaging setup.

### Comparison with a stereo microscope

**Fig. 4:**
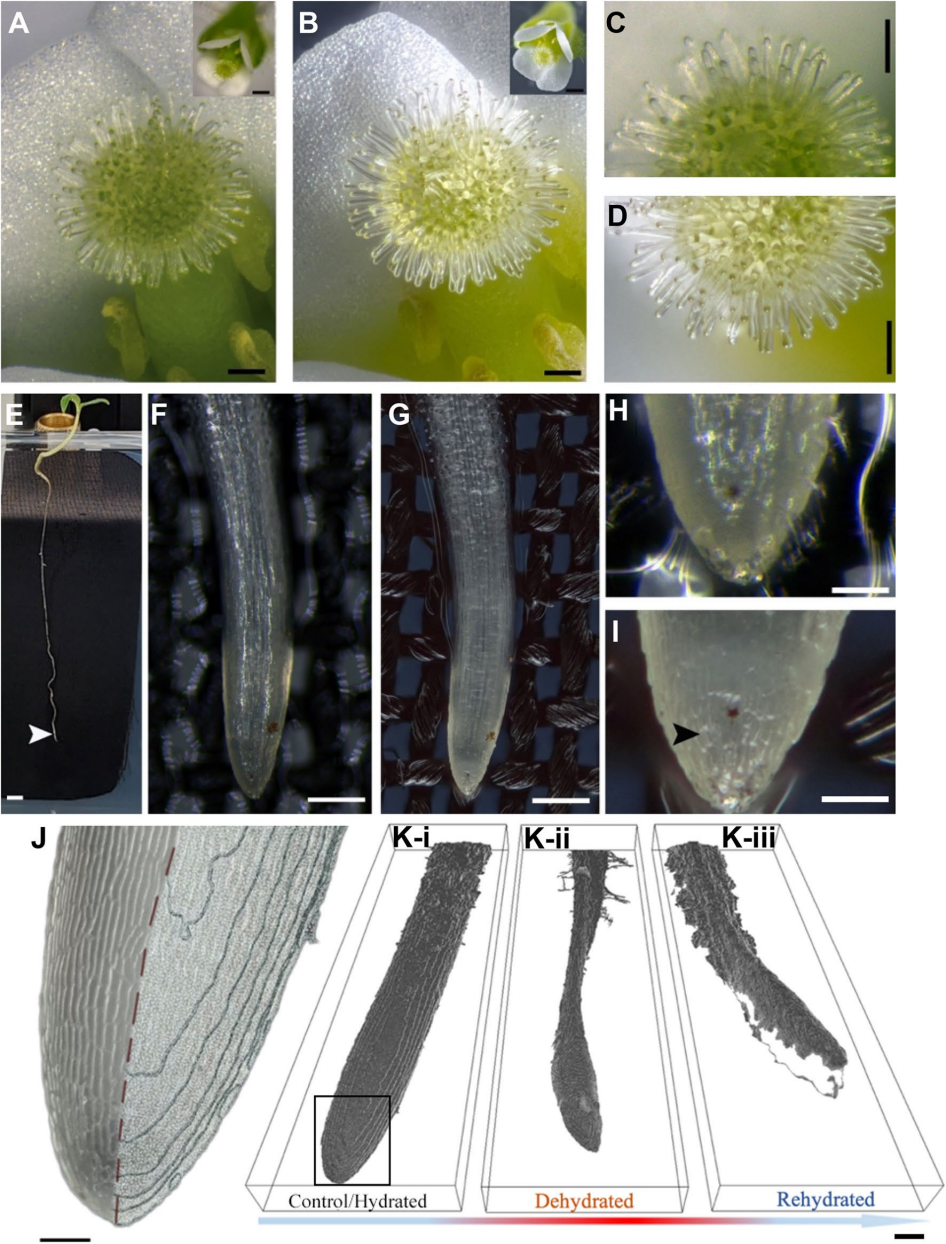
Real-world benchmarking of the Stemi 508 and imaging setup. (**A**-**B**) Arabidopsis flower imaged using the stereomicroscope and imaging setup at the native maximum of 4:1 and 5:1 magnifications, respectively (**A**-**B** right-hand corner); (**A**-**B**) are 7 × digitally enlarged crops highlighting the details from the right-hand corner full images. A close-up comparison in **C** and **D** depicts the stigmatic papilla imaged using both stereomicroscope and imaging setup, respectively. The snap in **D** is the same stack as in **B** but using only 12 images so that only the stigma is in focus (*N* = *5*). Similarly, we compared these two imaging systems using a tomato root (320 μm in diameter at the elongation zone) placed in a small acrylic plate with agar and an interface of dark fabric **E**. **H** root tip enlarged 10 × from **F** obtained using the stereomicroscope. Likewise, **I** 7 × enlarged snap of **G** shot using the imaging setup (*N* = *3*). **J** surface triangulation using the root profilometry methodology on a root tip enlarged from (**K**-i, control/hydrated). (**K**-i, ii, and iii) datasets represent one full rehydration cycle on the same root illustrated by the arrow below, starting from a hydrated state (**K**-i), to a dehydrated one (**K**-ii), revealing that the shape distortions occurred due to disturbance in the cellular hydrostatic equilibrium. After rehydrating (**K**-iii) the root, a partial recovery of the original shape of the root is shown (*N* = *10*). *N,* number of specimens analyzed. Scale bars: **A**, **B**, **C**, and **D** 0.1 mm, **E** 2.5 mm, **F** and **G** 0.25 mm, (**H**), **I** and **J** 0.05 mm, **J** and **K** 0.1 mm

To compare the system’s optical resolution and image quality, we imaged an Arabidopsis flower (45-days-old) and tomato roots (12-days-old) and compared them with the Zeiss Stemi 508 8:1 stereomicroscope images (Fig. 4A to I). We found that, at its maximum 4:1 magnification (due to the 0.5× demagnifying camera adapter), the Zeiss Stemi 508 stereo microscope had a FOV of approximately 1.44 × 1.08 mm. At its maximum magnification of 10:1 using the teleconverter, the proposed system still had a FOV eight times larger than the Zeiss Stemi 508 at its maximum 4:1 magnification. Likewise, the stereo microscope had a FOV of 11.61 × 8.67 mm at the minimum magnification, covering an area six times smaller than that from the imaging setup (Fig. 4I). In addition, the image sensor in the AxioCam 105 had a resolution of 4.92 megapixels (2560 × 1920 pixels). In contrast, the image sensor in the imaging setup had more than tenfold the number of pixels (50.6 megapixels or 8688 × 5792 pixels).

We demonstrated that we could achieve better image quality than the stereomicroscope (Fig. 4, A and B) by adequately lighting the specimens. The ability to perform focus stacking from snaps taken with z-axis steps of 10 μm at 10:1 magnification produced detailed images of the Arabidopsis flower (45-days-old) and tomato root, as depicted in Fig. 4. The superior performance and dynamic range were also visible in the noticeably cleaner images with less noise, chromatic aberration, and interfering reflections from specular surfaces (Fig. 4C and D).

### Monitoring dynamic deformation of the root surface

The focus stacking capabilities and higher resolution obtained by the imaging setup prompted us to create a framework for acquiring the surface profile of living roots during states of dehydration and rehydration (Fig. 4J and K). Using subsequent stacks of images taken at different times, we reconstructed a time-resolved outer surface of roots using the prescribed depth map given by the profilometry stack. This combination allowed us to acquire a dynamic deformation of the 3D models to study full-field displacement on the surface, providing the basis for implementing a brand new 4D imaging technique of roots in vivo (Additional file 7: Movie S6).

### Expanding the system to a multiplex design

**Fig. 5:**
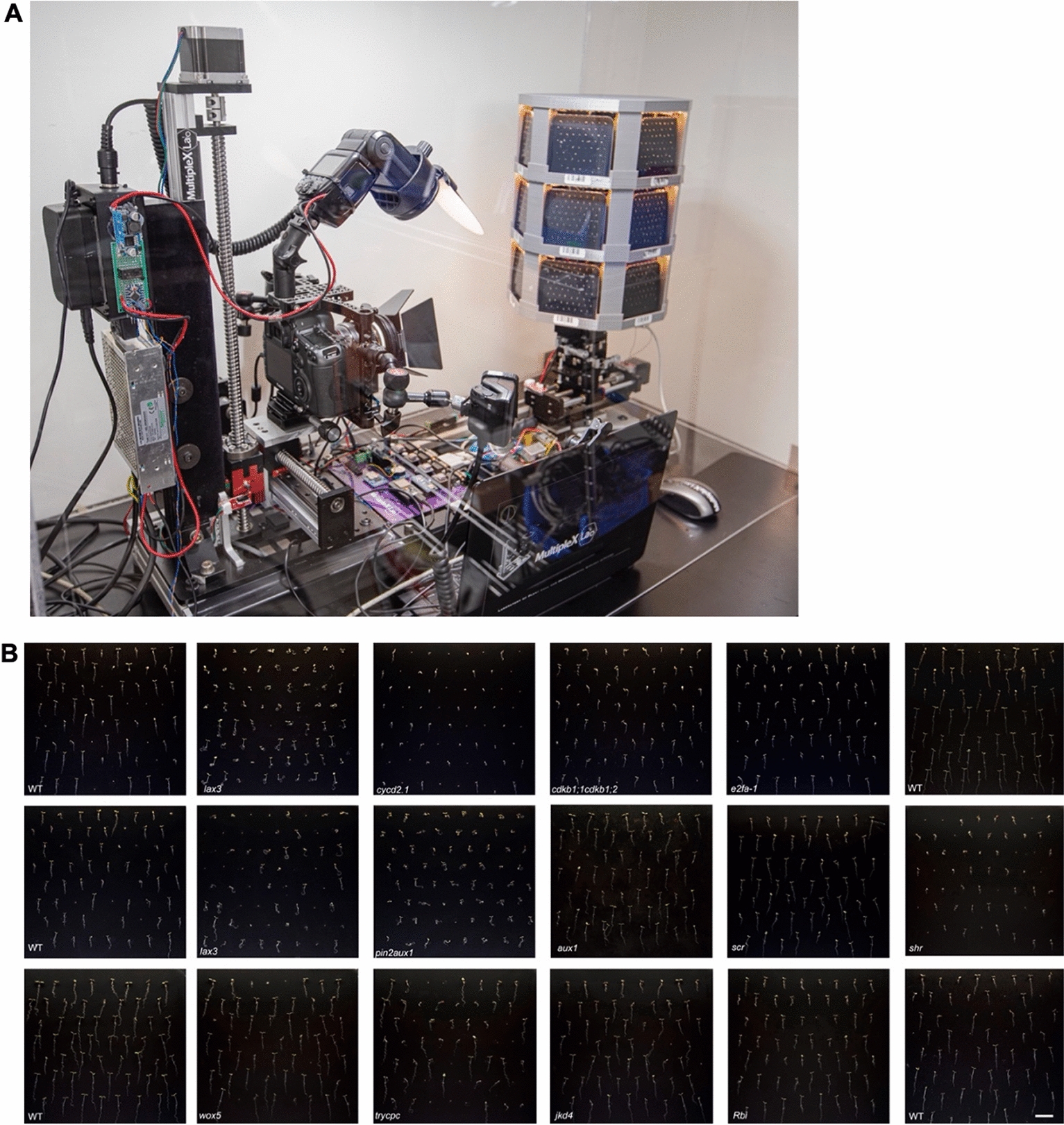
Toward high-throughput root imaging. **A** Multiple**XL**ab monitors 18 plates, each containing 64 Arabidopsis seeds for 4 days, and **B** collage image showcasing the frame for each plate at the end of 3 days of monitoring. Nonlinear adjustments were applied using a custom preset in Photoshop to even out the brightness of each image vertically. Scale: 1 cm. *N* = *1152* of seeds analyzed per run

Our single-plate imaging setup allowed us to acquire high-resolution images (Additional file 8: Movie S7). Next, we sought to convert the setup into a multiplate imaging platform; we named the resulting system Multiple**XL**ab (Additional file 1: Fig. S3, A to E). To build Multiple**XL**ab, we designed and 3D-printed a carousel stage that allows the user to load up to 18 plates at a 90° angle (Fig. 5). Multiple**XL**ab enables autonomous micropositioning of multiple plates relative to the camera. The device can be preprogrammed and independently operated using the onboard control systems. Alternatively, it can provide full functionality using the computer software Multiple**XL**ab Control Center UI (Additional file 1: Fig. S3E). For example, it can allow the end user to track the plate images tagged with a QR or barcode to control the lighting; acquire images on demand; and perform device calibration, focus stacking, and proper allocation of image files acquired in large numbers into labeled folders linked by the QR code.

### High-throughput imaging and data analysis

**Fig. 6:**
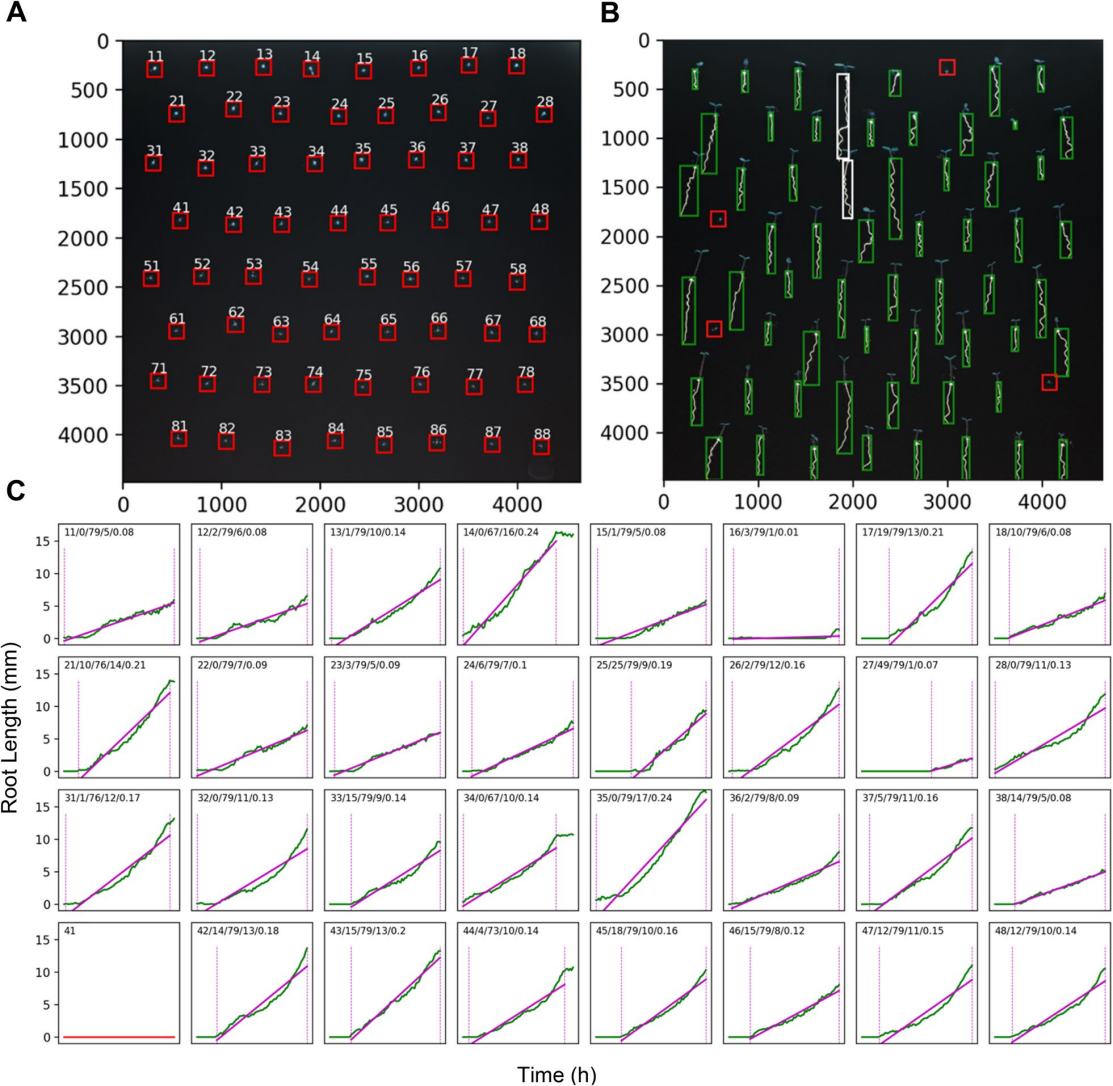
Germination dashboard. **A** Wild-type (WT) Arabidopsis seed detection based on SeedNet; **B** initial overlapping of bounding boxes from neighboring roots at 67 h; and **C** plots of individual root lengths from germination to the last viable timepoint for measurement—only half of the seeds are depicted here, see Additional file 1: Fig. S9 for the entire set. The group of five numbers on top of the plots represent the (i) seed/root ID (row, column), (ii) timepoint in which germination began (in hours), (iii) timepoint for the last measured timepoint (in hours), (iv) root length (mm) at the last viable timepoint, and (v) growth rate of roots (mm/h). The red horizontal line in seed ID 41 indicate nongermination. Scale: **A** and **B** 58.4 pixels/mm. *N* = *64*. *N* is the number of plants used in this study. Ten biological replicates were performed using this system

Multiple**XL**ab enables autonomous micro-positioning of multiple plates relative to the camera and can detect seeds, follow their germination, and track root growth. The system automatically exports the temporal information as a graphical germination dashboard (Fig. 6) accompanied by a datasheet file containing all data throughout the growth cycle. We developed an image processing pipeline combining traditional computer-vision algorithms with deep learning to achieve this. At its core, the pipeline relies on two deep learning models for image segmentation: SeedNet for finding seed pixels and RootNet for finding root pixels. The pipeline starts by analyzing the first frame and finds the seed pixels using the SeedNet model. The pipeline uses the OpenCV connected component algorithm to locate the individual seeds [20]. The connected components analysis returns the bounding boxes for each seed instance. The pipeline employs RootNet to locate the root pixels in each frame and expands the bounding boxes as the roots grow using the connected component algorithm. The pipeline determines bounding boxes for each root in each frame. Then, using the skeletonization algorithm implemented in the scikit-image processing library [21], the pipeline calculates the root length of each plant across the time series.

Finally, we used this information to plot root length versus time for each plant in a growth cycle. We fitted a line to the plot section, starting with the germination and ending with either an overlap between neighboring roots or the termination of the time series. Then, we calculated the growth rates as the slope of the fitted line.

### Scores of the deep learning models

The F1 scores (harmonic mean of precision and sensitivity) for SeedNet and RootNet on the test set were 0.8048 and 0.7395, respectively. The mean absolute percentage error on the root length measurements using long wild-type (WT) roots for validation was 4.18% (*N*=*12).*

### Implementing MultipleXLab for high-throughput phenotyping

**Fig. 7:**
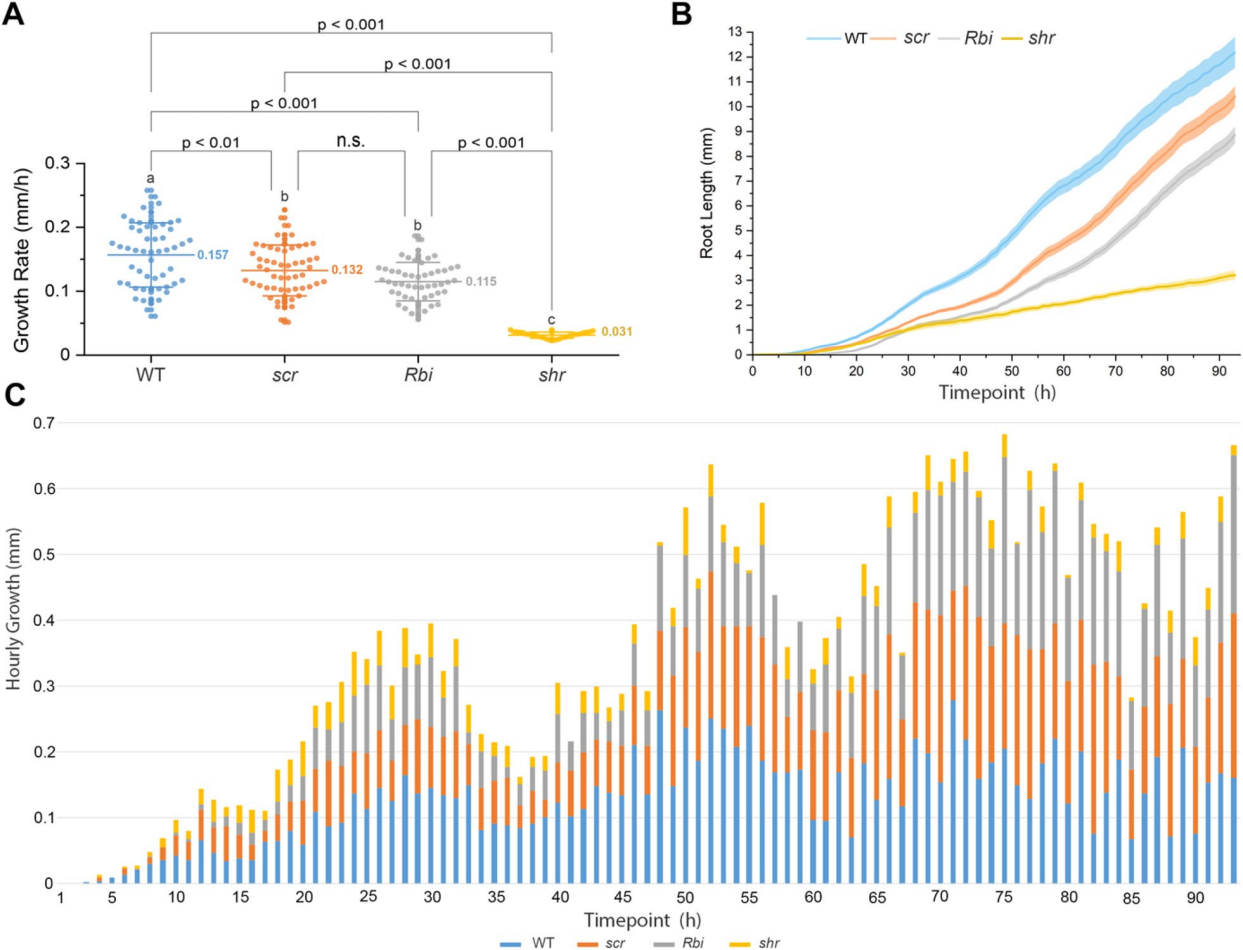
Root stem cell regulator mutants. Germination and growth rate scoring based on the analysis of variance and Tukey test. The wild-type (WT) has the highest growth rate, followed by *scr* and *Rbi*, and *shr* exhibited the lowest growth rate (mm/h). The growth rate of each root (datapoint) is calculated as the slope of the fitted line for the entire growth cycle. The number (*N*) of roots measured in each group of mutants for the WT, *scr*, *Rbi*, and *shr* were 53, 55, 51, and 28, respectively (total *N* = *187*). In **A**, the *y*-axis represents the growth rate (mm/h), and the *x*-axis represents different mutants. In **B**, the *y*-axis represents the root length (mm), and the *x*-axis indicates the duration of root growth in hours. **C** represents the hourly growth rate throughout the growth cycle; the *x*-axis represents the time point of growth of each mutant. Colored bars indicate the mutants analyzed. Additional file 1: Fig. S10A complements **C**. Overlapping between roots first started at 49 h in the WT group. Three biological replicates were tested using this system

**Fig. 8:**
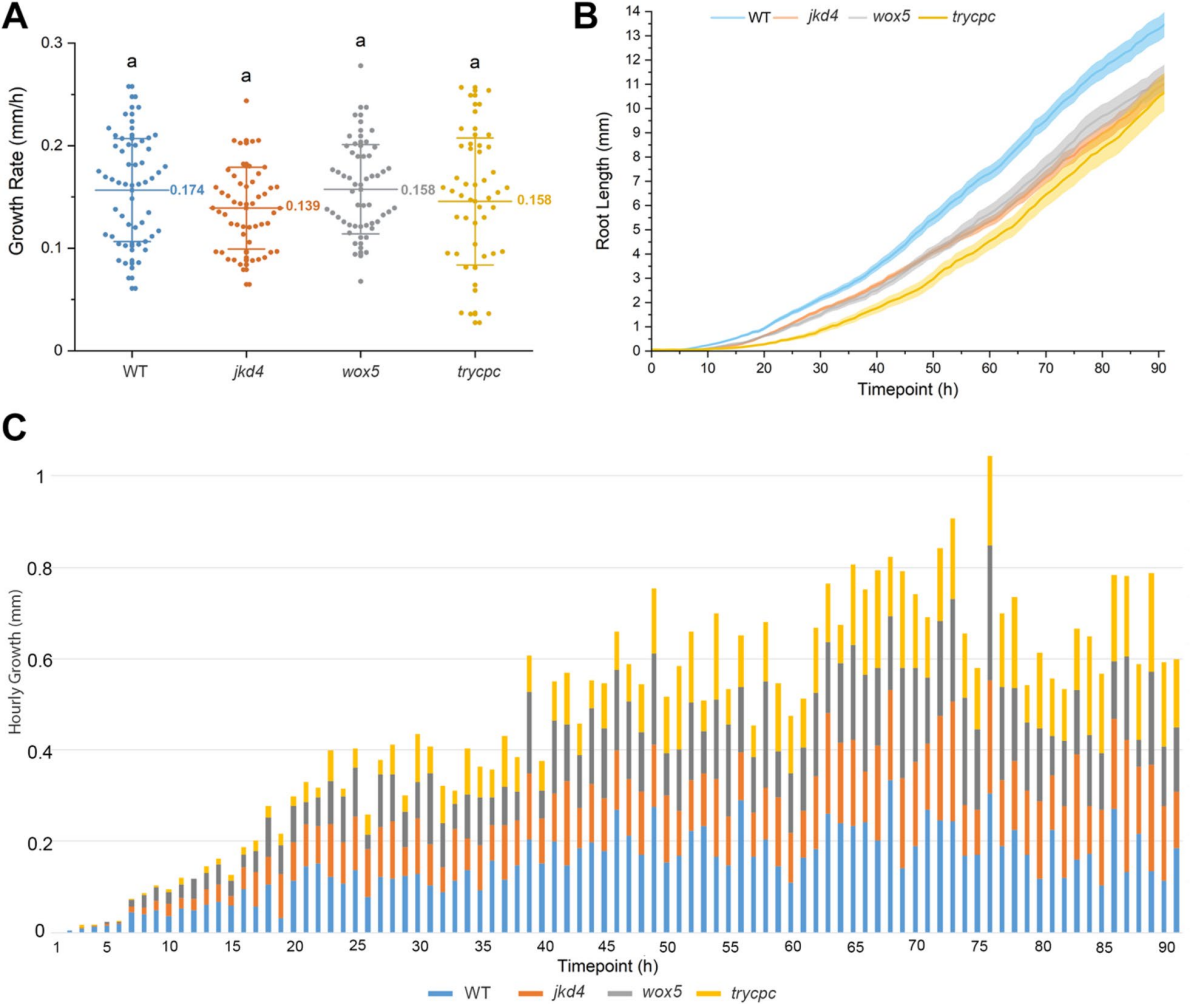
Root stem cell and patterning mutants. Germination and growth rate scoring based on the analysis of variance and Tukey test. No statical difference was detected for mutants *jkd4*, *wox5*, and *trycpc* compared to the wild-type (WT) mean growth rate (mm/h). The number (*N*) of roots measured in each group of mutants for the WT, *jkd4*, *wox5*, and *trycpc* was 56, 53, 52, and 45, respectively (total *N* = *206*). In **A**, the *y*-axis represents the growth rate (mm/h), and the *x*-axis represents the time point of growth of each mutants. Colored bars indicates the mutants analyzed. In **B**, the *y*-axis represents the root length (mm), and the *x*-axis indicates the duration of root growth in hours. **C** represents the hourly growth rate throughout the growth cycle; the *x*-axis represents the time point of growth of each mutants. Colored bars indicate the mutants analyzed Additional file 1: Fig. S10B complements **C**. Overlapping between roots first started at 64 h in the WT group. Three biological replicates were tested using this system

**Fig. 9:**
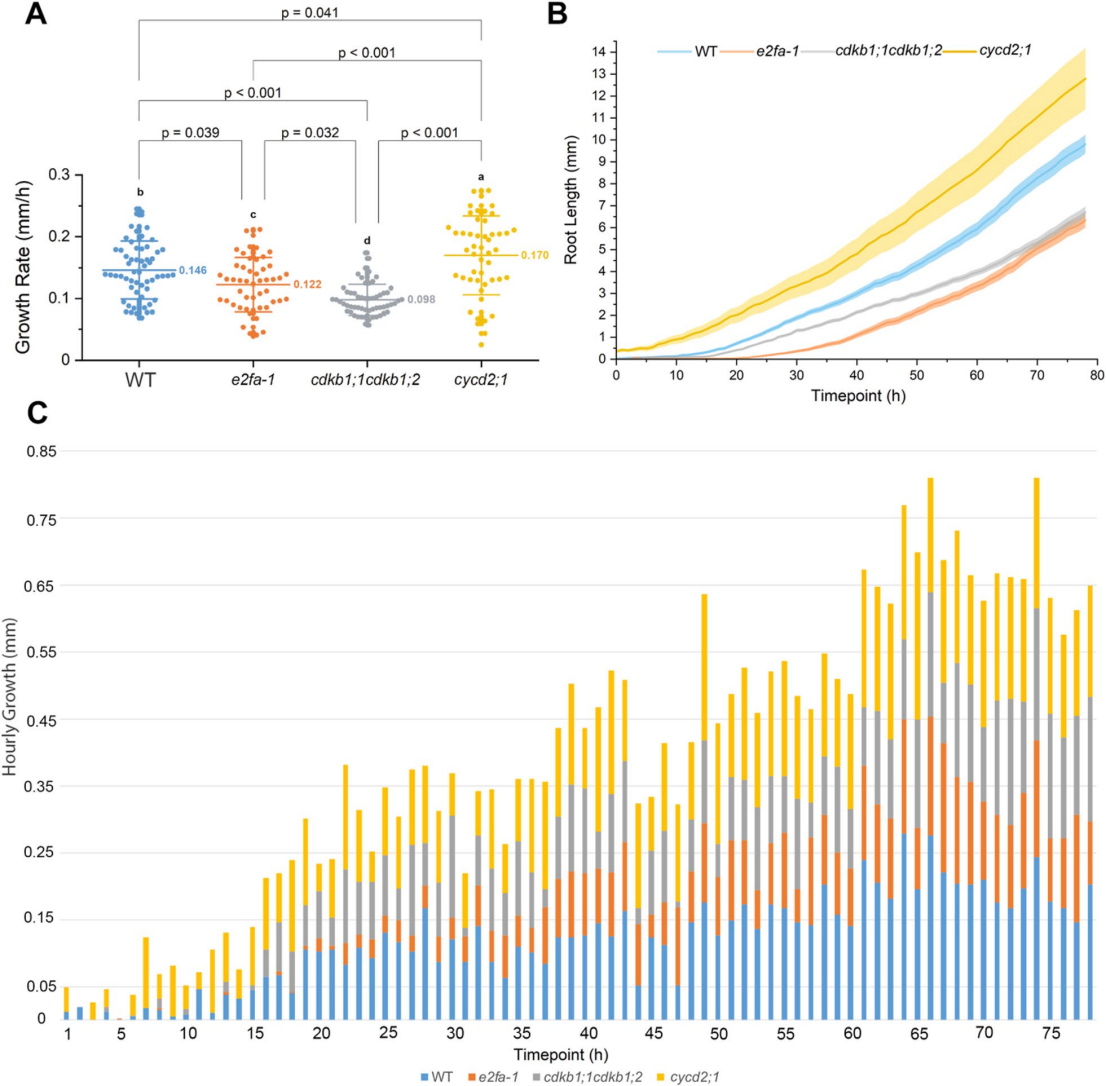
Cell cycle mutants. Germination and growth rate scoring based on the analysis of variance and Tukey test. The *cycd2;1* had the highest growth rate, and *e2fa-1* and *cdkb1;1cdkb1;2* had the lowest. The number (*N*) of roots measured in each group of mutants for the wild-type (WT), *e2fa-1*, *cdkb1;1cdkb1;2*, and *cycd2;1* was 60, 51, 59, and 49, respectively (total *N* = *219*). *N* is the number of plants used in this study. In **A**, the *y-*axis represents the growth rate (mm/h). *x*-axis represents different mutants. In **B**, the *y*-axis represents the root length (mm), and the *x*-axis indicates the duration of root growth in hours. Colored bars indicates the mutants analyzed **C** represents the hourly growth rate throughout the growth cycle; the *x*-axis represents the time point of growth of each mutant. Colored bars indicate the mutants analyzed Additional file 1: Fig. S10C complements **C**. Overlapping between roots first started at 53 h in the *cycd2;1* mutants. Three biological replicates were tested using this system

**Fig. 10:**
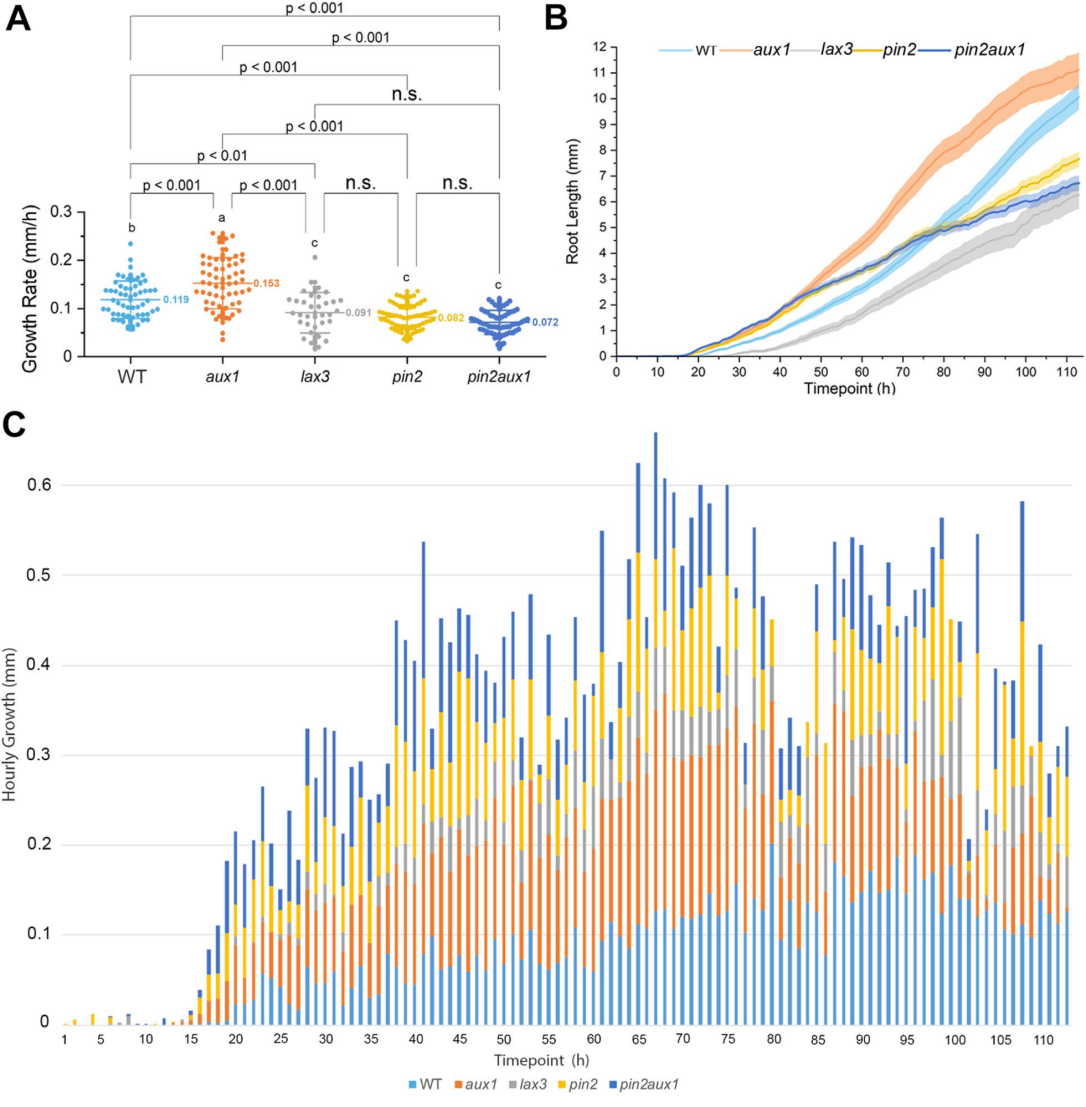
Auxin transport mutants. Germination and growth rate scoring based on the analysis of variance and Tukey test. The *aux1* had the highest growth rate, and the mutants *lax3*, *pin2*, and *pin2aux1* had the lowest growth rates (mm/h). The number (*N*) of roots measured in each group of mutants for the wild-type (WT), *aux1*, *lax3*, *pin2,* and *pin2aux1* was 57, 60, 34, 64, and 61, respectively (total *N* = *276*). In **A**, the *y*-axis represents the growth rate (mm/h), and the *x*-axis represents different mutants. In **B**, the *y*-axis represents the root length (mm), and the *x*-axis indicates the duration of root growth in hours. **C** represents the hourly growth rate throughout the growth cycle; the *x*-axis represents the time point of growth of each mutant. Colored bars indicate the mutants analyzed. Additional file 1: Fig. S10D complements **C**. Overlapping between roots first started at 67 h in the *aux1* mutants. Three biological replicates were tested using this system

To test the potential of Multiple**XL**ab, we monitored seed germination and root outgrowth of several developmental, auxin transport and cell-cycle mutants (Figs. 7, 8, 9 and 10). For the root-developmental mutants, we used the stem cells and asymmetric cell division factors *scarecrow* (*scr*), *shortroot* (*shr*), *Retinoblastoma related 1 (RBR1) RNA interference (Rbi)*, and *jackdaw* (*jkd*); the quiescent center function regulator *Wuschel-related Homeobox 5 (wox5*); and the root hair patterning double mutant *triptychon caprice* (*trycpc*) [22–27]. We found that WT plants have a germination index ranging between 92 and 100% (Tables 1, 2, 3, 4). Among these mutants, only *shr* and *trycpc* exhibited a lower germination index of 74% and 80%, respectively (Tables 1 and 2).

**Table 1:** Germination index and growth rate of root stem cell regulator mutants

Genotype	Germination index (%)	Growth rate (mm/h)	*N*
WT	96	0.15672^a^	53
*scr*	98	0.13240^b^	55
*Rbi*	98	0.11503^b^	51
*shr*	74	0.03135^c^	28

Tukey; different letters indicate that individual means are significantly different (*p* < .05)

**Table 2:** Germination index and growth rate of root stem cell and patterning mutants

Genotype	Germination index (%)	Growth rate (mm/h)	*N*
WT	100	0.17367^a^	56
*jkd4*	95	0.13914^a^	53
*wox5*	96	0.15751^a^	52
*trycpc*	80	0.14568^a^	45

Tukey; different letters indicate that individual means are significantly different (*p* < .05)

**Table 3:** Germination index and growth rate of auxin transport mutants

Genotype	Germination index (%)	Growth rate (mm/h)	*N*
WT	92	0.11854^b^	57
*aux1*	94	0.15267^a^	60
*lax3*	53	0.09149^c^	34
*pin2*	100	0.08241^c^	64
*pin2aux1*	100	0.07152^c^	61

Tukey; different letters indicate that individual means are significantly different (*p* < .05)

**Table 4:** Germination index and growth rate of cell-cycle regulator mutants

Genotype	Germination index (%)	Growth rate (mm/h)	*N*
WT	94	0.14602^b^	60
*e2fa-1*	80	0.12230^c^	51
*cdkb1;1cdkb1;2*	100	0.09782^d^	59
*cycd2;1*	78	0.16982^a^	49

Tukey; different letters indicate that individual means are significantly different (*p* < .05)

Next, we tested the auxin transport mutants using the auxin influx carriers *aux1* and *lax3*, the auxin efflux carrier *pin2*, and the double mutant *pin2aux1* [28–30]. We found that *lax3* had a lower germination index (53%) compared to WT (92%) and other auxin transport mutants (Table 3).

We also included the cell-cycle regulators *e2fa-1*, the cell-cycle-dependent kinase double mutants *cdkb1;1cdkb1;2*, and a mutant of the Arabidopsis *D-Type Cyclin CYCD2;1* the *cyclin d2;1 (cycd2-1)* [31–33]. We found that *e2fa-1* and *cyclin d2;1* had a lower germination index than the WT at 78% and 80%, respectively, whereas other cell-cycle mutants were similar to the WT (Table 4).

Next, we calculated the growth rate by monitoring the germination and root growth of individual seeds and roots hourly for each mutant. To this end, we combined time-lapse imaging with image segmentation based on deep learning in root systems (Additional file 9: Movie S8). This approach allowed us to evaluate and extract differences in growth dynamics of thousands of samples simultaneously and precisely detect the germination initiation timepoint. We found that *shr* mutants have a slower growth rate (Fig. 7), with an average growth rate (AGR) of 0.03135 mm/h in *shr* compared to 0.15672 mm/h in the WT.

Hourly image acquisition allowed us to evaluate the growth rate of each mutant. We found that the WT had the most significant growth rate during the initial 48 h (AGR = 0.15672 mm/h for the entire cycle), while *scr* and *Rbi* grew more slowly (AGR = 0.1324 and 0.11503 mm/h, respectively) in the beginning and caught up after 65 h, exhibiting growth rates similar to WT at the end (Fig. 7). In addition, *shr* mutants displayed a slow and constant growth (AGR = 0.03135 mm/h) with the lowest growth rate of all the mutants. Despite not displaying statistically different root length phenotypes compared to the WT, we found that the double mutant *trycpc* had a relatively constant growth rate during the first 30 h and was revealed to be slower throughout the entire cycle (AGR = 0.14568 mm/h). In contrast, the *wox5* growth was slow (AGR = 0.15751 mm/h) due to delayed germination, as initial growth was observed 4 h after the WT (Fig. 9). Finally, *jkd* also displayed a slight delay in growth during the first 5 h but gained speed to reach WT’s growth rate at the end (AGR = 0.13914 mm/h).

Subsequently, we followed the growth of mutants involved in auxin transport. These mutants are challenging to evaluate because of their agravitropic phenotype, causing the root to curl and obstruct certain parts under itself, leading to discontinuity and artifacts during measurements; hence, it may introduce a biased result for the root length measurements (Additional file 1: Fig. S4, A to C). We used the skeletonization algorithm to measure the root segment summation length within the root domain prescribed by the bounding box (Additional file 1: Fig. S4C) to mitigate this challenge. We found that *lax3* has significantly shorter root growth than the WT (AGR = 0.09149 and 0.11854 mm/h, respectively). The double mutant *pin2aux1* was similar to *lax3*. We also analyzed the efflux carrier mutant roots and found that *pin2* (AGR = 0.08241 mm/h) has significantly shorter roots than the WT (AGR = 0.11854 mm/h). The roots of *pin2aux1* (AGR = 0.07152 mm/h) double mutants exhibited a phenotype similar to *pin2*, indicating that *pin2* is epistatic t*o aux1* in term of root growth. While analyzing the growth dynamics of the auxin mutants, we noticed that the WT controls had a 7-h delay in growth (Additional file 10: Movie S9). Moreover, *pin2* had the fastest germination, whereas *pin2aux1* and *lax3* had the slowest growth rate of the auxin mutants tested in this study (Fig. 10).

The cell-cycle mutants displayed an interesting growth rate. The analysis revealed that *e2fa-1* (AGR = 0.1223 mm/h) has a slightly slower root-growth rate than the WT (AGR = 0.14602 mm/h). We also observed large fluctuations, especially during the early time points. For example, *cycd2,1* seemed to have a faster growth (AGR = 0.16982 mm/h), which stabilized after 24 h, and *cdkb1,1;cdkb1;2* (AGR = 0.09782 mm/h) germinated faster. However, its growth halted and became slower until 15 h, after which the growth speed became similar to that of the WT (AGR = 0.14602 mm/h; Fig. 10). Finally, the *e2fa-1* mutant behaves similarly to *cdkb1,1;cdkb1;2* but resumed normal growth after 36 h.

## Discussion

Our imaging setup provides a solution to the long-standing challenge faced by biologists when capturing images of dynamic processes in large samplings of living organisms. During growth, organs change continuously in shape and size and, particularly in the case of plants, are formed throughout an organism’s entire life cycle. A wide variety of platforms have been designed for different biological systems to follow plant growth [10, 33–38], digitize and 3D model insects [39], and employ hybrid microscopy [16] in addition to other setups for 3D automated imaging [13] and photogrammetry [40]. We demonstrate that Multiple**XL**ab, with its high-range 3D-printed carrousel (Additional file 1: Fig. S5), provides a solution for a wide range of applications by resolving features at a high magnification and resolution (Additional file 1: Fig. S5, C and D).

We used the proposed system to monitor the germination index and root-growth rate for thousands of Arabidopsis seeds. The ability of the system to image and quantify the growth rate of several samples simultaneously in an hourly manner enabled us to extract new phenotypes, such as the slower growth of *Rbi* and *lax3* mutants and the faster growth rate for *cdkb1,1;cdkb1;2*. The system can also dissect differences between mutants and to evaluate the seed quality in different seed batches. The experiment using auxin mutants is a good example, where the delayed WT growth is most likely related to the seed batch.

Based on cameras/lenses designated for the consumer market, the proposed system is more versatile than microscopes for simultaneously resolving macro- and micrometric structures, primarily due to its high-density pixel sensor and additional flexibility in lighting the specimens. Moreover, in the proposed system, the camera can be detached from the rail and repurposed for general photography. The various configurations proposed in this setup allow reasonable working distances between the outer-filter element on the lens and specimen, which permits proper illumination. Additionally, the optics can be easily stacked, and the widely available camera/lens mount adapters available for the Canon EF bayonet allow for fast and straightforward lens swaps, including infinity-corrected microscope objectives (Additional file 1: Fig. S6, G and H). These unique features allow the user to alternate between a reduced FOV for maximum magnification or a larger FOV for optimized magnification, offering more flexibility to fit a wide range of specimen dimensions. Most importantly, this setup has the advantage of allowing us to adapt inexpensive optics from innumerable different focal lengths, including the adapted legacy lenses, making this type of imaging system more versatile than most commercially established benchtop lab systems.

Creating surface topography images allows the proposed system to have unique applications, such as the nondestructive surface mapping of a living organism. This feature becomes relevant when dealing with living organisms subjected to changes in their surrounding environment (e.g., plant roots subjected to drought, nutrient depletion, and changes in soil composition) or reacting to continuous threats from pathogens, such as fungus, soil nematodes, and insect herbivores. Using this imaging setup, we monitored changes over time in the 3D structure of the root surface during drought, allowing us to create a surface representation of the root topology (Fig. 4J and Additional file 7: Movie S6). We predict that this methodology can be extended to other organs, plant species, and organisms. Combining the multiplate stage holder with preprogrammed cycles enabled us to continuously monitor and grow multiple specimens indoors and precisely control the lighting used for plant growth using a specific daily cycle [16 h on and 8 h off]. The automation speed for acquiring multiscale images, stitching, and stacking makes Multiple**XL**ab a versatile and powerful CNC microscope.

In addition, we implemented an image segmentation pipeline powered by deep learning to facilitate and accelerate the analysis of numerous multidomain images. This approach has recently received much attention because it provides an attractive solution for fast detection and measurement tasks in complex applications [41–45], providing a basis for automatically measuring phenotypic traits. These tools can perform supervised and unsupervised root segmentation using convolutional neural networks based on classical deep learning architectures. These new solutions help automate data extracted from large datasets, such as those generated in this study. Although these tools are valuable and attractive to use, it is essential to carefully design a well-trained and robust pipeline that allows the user to obtain reliable measurements that are representative of the phenotype in question, in our case it required manual labeling of thousands of seed/roots (Additional file 1: Fig. S7, A to D). In agravitropic roots, the high temporal and spatial resolution of image acquisition every hour provides a basis for tightly expanding bounding boxes around roots. This resolution allows the root pixels inside each bounding box to be computed even when root components may discontinue during development for several reasons, such as obstruction of roots into itself or by the hypocotyl, seed coat, or cotyledons. In a few cases, the system failed to detect and track certain seeds into developed roots due to a high degree of seed displacement due to agar shrinkage by drying (Additional file 1: Fig. S4D). Therefore, the computer-vision interpreter and actuator of the bounding box expansion have room for improvement (e.g., using an analysis of component connectivity with more flexible expansion parameters). Furthermore, improving the segmentation model by extending the training using more edge cases can improve detection and tracking in future datasets.

High-throughput phenotyping technologies powered by artificial intelligence are now important tools for advancing genetic gain in breeding programs [2] and assessing the effects of natural variation or treatments on plant development [46]. This study demonstrates that computer-vision analysis permits autonomous image processing in pipelines designed to analyze gravitropism and explore temporal micro-morphometrics in overwhelmingly large multiscale datasets. These high-throughput analyses are fundamental steps to alleviate bottlenecks in precision agriculture in crop phenomics (Additional file 1: Fig. S8). Some of these limitations are data storage and functional modes to evaluate root-phenotypic metrics with multiple and distinct traits quantitatively [47].

A downside of the proposed system is that is lacks the capacity to extend the duration of experiments. In the current device we can monitor root growth continuously only for a limited time window (maximum 4 to 5 days) when using agar plates because of the dehydration of agar over long experiments. Another limitation is the water condensation on the lid that we bypassed by removing, but this sometimes caused fungal contamination. Furthermore, when using Arabidopsis, the lighting responsible for plant development can get limited in certain seedlings due to the plating scheme (8 rows x 8 columns). Upon development of large cotyledons on the top rows around the fourth day after plating, it can cause the bottom rows with seedlings to get light-deprived, causing etiolation.

Some minors limitations of this imaging setup is the need to use band filters in front of the lenses to acquire images in specific spectrum ranges (ultraviolet-visible-infrared), which may affect the working distance and the flexibility to illuminate the specimens at high-magnifications. Furthermore, the lack of auto-focus capabilities in the main optical array prevents this setup to automatically pull focus in respect to the specimen, thus requiring the operator to determine initial parameters of focal length/distance in the Control Center program. Additionally, large file sizes and computationally expensive tasks during post-processing operations may require a high-end computer/laptop to handle the data processing, depending on the duration of the time series.

## Conclusions

Multiple**XL**ab is a mobile, modular phenotyping platform for automatically monitoring seed germination and root growth. It can be used in multiple applications by plant biologists, the seed industry, crop scientists, and breeding companies. First, the system can be used to screen seed vigor (germination and viability), allowing farmers and seeds companies to test the newly received seed batches and evaluate the viability of seed stocks. Second, it can be used to screen for particular phenotypes resulting from ethyl methane sulfonate screens, including germination rates, root length, and gravitropism. Third, it can screen for mutants or cultivars under various conditions, assessing their response to multiple stresses, such as salinity and nutrient deficiency. Finally, it can monitor the growth response to growth-promoting substances and beneficial bacteria or the resistance to pathogens.

## Materials and methods

### Sample preparation

#### Arabidopsis samples

Arabidopsis seeds were gas-sterilized using 100 mL of sodium hypochlorite (commercial bleach) supplemented by 3 mL of 37% HCl for at least 2 h. Seeds were embedded in agarose and stratified overnight at 4 °C before sowing on half Murashige and Skoog (MS) plates. Seeds were sown in square Petri dishes with a distance of 1 cm between each other. Plates were imaged from germination to early root growth (up to 5 days) under a photosynthetic photon flux (PPF) of 180 µmol m^-2^ s^-1^ using an 18/6-h lighting cycle.

#### Tomato samples

To test the imaging setup in the greenhouse (23°C; 70% relative humidity [RH]), we conducted an experiment to observe the effects of wounding on the roots of tomatoes (*Solanum lycopersicum*). We planted tomato seeds on top of a nylon mesh between the soil and polystyrene plates, which resembled a small rhizotron. This small 12.5 × 10 × 5 cm Petri dish rhizotron was cut at the top to allow the plants to grow out, with the planted seeds at about ¾ of the plate height at an angle of −45°. This configuration, combined with a nylon mesh interface between the seeds and soil, allowed the roots to grow only on the mesh, facilitating the monitoring of specific conditions in the root system architecture during long-term experiments.

#### Tomato root regeneration

Tomato roots were grown for 4 days on soil, and then the meristem was excised using dental microneedles. The root growth was continuously monitored for a few days until full regeneration was detected.

#### Dehydration experiment

Tomato seedling with a few millimeters in length [3 days after sowing] was transferred onto a wet mesh sitting directly on the top of a Petri dish with no agar for the first imaging stage (control/hydrated). As the moisture in the mesh dries in about 45 minutes (25 °C; 60% RH), the root begins to dehydrate, shrink, and deform (second stage − dehydrated). After the root was dehydrated, we wet the mesh for 10 min to rehydrate the root and capture the third and last stage. The stacking operation of 70 shots took approximately 15 minutes to be acquired in each stage.

#### Customized mini-rhizotron

The mini-rhizotron system was set up using a standard transparent polystyrene square Petri dish cut open on one side. It was filled with soil and entirely covered by a 100 μm resolution nylon mesh (Sefar Nitex 03-100/44; Additional file 1: Fig. S1) to prevent the roots from growing into the soil and remain visible on the mesh surface to facilitate imaging.

#### Building the imaging system

The initial imaging setup consists of a modified digital camera, the Canon 5DSr SLR (Canon Inc., Tokyo, Japan), with a 50.1-megapixel full-frame sensor (36 × 24 mm). The camera is attached to a Canon MP-E 65 mm f/2.8 1-5x lens stacked on a 2x teleconverter (Vivitar Series 1) mounted on a vertical/horizontal motorized rail system (WeMacro, Shanghai, China; Fig. 1, A to H, Additional file 1: Table S1), Multiple**XL**ab’s simplest configuration. A step-by-step assembly of the single-plate imaging setup is presented in Additional file 8: Movie S7. The expanded version achieved by Multiple**XL**ab (Fig. 4J) is also equipped with a Canon EF 40 mm f/2.8 STM lens stacked on a 2:1 teleconverter (Vivitar Series 1). This setup provides a unique 28° angle of view at 30 cm away to cover the entire view (150 cm2) of the Petri dish and permits proper lighting and larger magnification of 0.24x (0.18x without the teleconverter) at life-size, given by the 15 cm gain in the minimum focus distance. A set of band-pass filters, including infrared, visible light, and ultraviolet light, can be used in front of the lens element to narrow the spectrum of interest.

#### Lighting enclosure for high-resolution imaging using the single-plate setup

The imaging system was placed inside a large, illuminated lightbox (80 × 80 × 50 cm) that provides constant lighting for imaging, and it is used to assist in refocusing the optical system, as well as in video recording. Furthermore, a ring flash (Canon MR-14EX II) was attached to the lens, and two Speedlite flashes (Yongnuo YN600EX-RT II) that were diffused with a strap-on light softbox (15.2 × 12.7 cm) were used. Mounted on articulated arms (Manfrotto 244 Variable Friction Magic Arm, Cassola, Italy), these external flashes provided oblique light on the specimens inside the lightbox to assist in high-magnification imaging when high light intensity is required. We also used three 150 W E27 5500 K lightbulbs in the light modifiers outside the lightbox to provide constant fill light and facilitate sample alignment and focusing during imaging. These components are listed in Additional file 1: Table S1 and depicted in Fig. 1I and Additional file 8: Movie S7.

#### 3D-printing a multiplate carousel stage

First, the hexagonal 3D-printed stage design was conceptualized and designed using SketchUp (v.18.0.16976, Google LLC, Mountain View, California, USA). Then, it was rendered using Fusion 360™ (Autodesk®, Inc., Mill Valley, California, USA; Additional file 1: Fig. S5). The design file was exported to an STL format and converted into a printable file using the slicing software ideaMaker (Raise 3D, Irvine, California, USA) to set printing parameters on the raft base. The layer height was set to 0.2 mm, the infill to 15%, and two shells were used. The file was then loaded into the Raise 3D Pro 2 printer and printed in several sessions of 27 h for each stage level.

#### Building the MultipleXLab control system

The device is equipped with high-resolution stepper motors (Nema 17 or 23 Model 17HS15-1684S-PG5, 1.8° per step) in three linear actuators (400, 200, and 150 mm stroke) using ball screw (Fuyu Motion FSL40, Sichuan, China) and one rotary table (PX110, Beijing PDV Instrument Co., Ltd, China) driving the carousel, which also interfaces with two stacked goniometers to provide fine tilt adjustments in high-magnification applications to achieve parallelism with the vertical axis carrying the camera. The system can operate under preset lighting cycles (18/6 on/off) using built-in plant-growth lighting at 24 V, providing up to 400 µmol m^-2^ s^-1^ of PPF within a 10 cm distance. Using the 3D-printed carousel with three levels, we can tightly fit 18 plates simultaneously (Additional file 1: Fig. S3) and precisely lighten the plates with cross-polarized lighting using an array of Speedlight flashes covered with linear polarizer films (P100A-3Dlens, Taipei, Taiwan) working in conjunction with a circular polarizer (B+W MRC filter, Bad Kreuznach, Germany) on the Canon EF 40 mm f/2.8 STM lens stacked on a 2:1 teleconverter, effectively turning it into an 80 mm focal length lens.

The system is integrated using a custom printed circuit board with an ESP32 as the master microcontroller to control the lighting and the camera shutter release (Additional file 1: Fig. S3D). It also reads a program from an SD card to perform routine monitoring cycles in selected plates. Additionally, the ESP32 interfaces with an I2C port expander, enabling controlling and communicating with auxiliary sensors and actuators and a real-time clock module to synchronize the program timing and lighting cycles accurately. A slave microcontroller performed by an Arduino Nano acts as a liaison to the stepper motors, and it receives commands from the ESP32, completes stepper operations, and handles limit switches in the calibration step. The power consumption of the entire system is approximately 0.1 kWh.

The device can be preprogrammed and independently operated using the onboard control systems. Alternatively, the user can run the system using the Multiple**XL**ab Control Center UI software (Additional file 1: Fig. S3E). The software has features that allow the end user to perform device calibration and focus stacking, control the lighting, and acquire images on demand. Images corresponding to each plate were tagged with a QR or barcode to facilitate data acquisition and management. The images can be easily retrieved, allowing the proper allocation of numerous images into labeled folders linked by the QR code.

#### Imaging settings

We employed a set of different imaging settings during z-stacking, single-snap, and time-lapse. For each condition, as determined by sample size, geometry, and environment, we specifically tailored the lighting as needed by selecting different types of external lighting and light modifiers to create soft and diffused lighting around the specimens. For comparison, we also acquired images using the stereomicroscope Stemi 508 (Carl Zeiss Microscopy GmbH, Jena, Germany) coupled with a color CMOS camera (AxioCam 105 by Carl Zeiss Microscopy GmbH) using a camera adapter (Zeiss 60N-C 2/3 0.5X) operated using the manufacture’s ZEN lite imaging acquisition software.

Macrophotographs of plant organs were shot in raw CR2 format with a native resolution of 8688 × 5792 pixels, taken in 40-μm step increments in z-resolution at 5:1 magnification, f/2.8, 1/200 s, and ISO 100. These were combined with a 2:1 teleconverter turning the effective magnification to nearly 10:1 and two perpendicular Speedlight flashes optically triggered by one parallel ring light flash at 1/64 power mounted on the camera. The imaging parameters for the z-stacks were different for each specimen. The Thysanoptera in Arabidopsis leaves was imaged using a single exposure (i.e., no focus stacking) because the insect was continuously moving; therefore, we used a faster shutter speed of 1/2500 s and an aperture of f/5.6 without the 2:1 teleconverter. When capturing the static ladybird beetle (*Harmonia axyridis*) on Arabidopsis leaves, we employed focus stacking with a finer 20-μm stepping in z-resolution to counter the even shallower depth of field.

We used a flower as a specimen to assess the performance of the single-plate imaging setup versus a stereomicroscope. A stack of 21 (2560 × 1920 pixels) snaps was taken using the stereomicroscope by manually focusing through the entire depth of the visible parts of the flower. The image was obtained from 103 stacks (8688 × 5792 pixels) stepped in 10 μm in z-resolution at about 10:1 magnification, f/5.6, 1/100 s, and ISO 100, using two perpendicular Speedlight flashes optically trigged by a ring light flash mounted on the camera using the single-plate imaging setup; all set at 1/16 power. The close-up comparison of the stigmatic papilla was captured using the stereomicroscope and the proposed system. The final image was made from 13 stacked images obtained by manually focusing through the entire depth or radial thickness of the root using the stereomicroscope. The image obtained from the single-plate imaging setup was made using 12 images so that only the stigma was in focus. The entire datasets from time series acquired using the Multiple**XL**ab were taken at f/11, 1/10 s, and ISO 200, with external flashes set at 1/32 power.

Batch processing using Adobe Photoshop CC (20.0.5) was employed to handle the thousands of images generated by the Multiple**XL**ab device. Each raw image has a file size of around 50 MB. These raw images were treated using the Camera Raw Editor in Adobe Photoshop CC to apply a fixed preset that was created to even out brightness in the image, correct color temperature, and increase contrast. The output images were exported to digital negative format and aligned using the auto-align translation function through a batch process in Photoshop. Aligned frames from the same plate were center-cropped to a fixed size of 4639 × 4480 pixels, comprising the region of interest. Frames were exported to TIFF format using an automated batch process, and each TIFF frame had a final file size of approximately 30 MB. Time lapses and animations were rendered using Final Cut Pro 10.5.0 (Apple, Cupertino, California, USA).

#### Measuring pixel contrast between the imaging setup and stereomicroscope

The calculated theoretical maximum numerical aperture of the imaging setup was 0.09 at 1× magnification and 0.03 at 5× magnification. Subsequently, we determined the lateral resolution of the entire imaging setup based on the calculated modulation transfer function using an inexpensive 1951 USAF resolution target^5^ with negative and positive chrome patterns manufactured according to MIL-S-150A standards, measuring 63 × 63 × 2 mm and containing the entire group and elements from 0 to 7, indicating a minimum and maximum of 1 to 228 lp/mm, respectively.

The resolution target was imaged at 4:1 magnification using the stereomicroscope at its maximum brightness illumination and auto-exposure based on a selected area of the negative chrome pattern on the mask. For the 5:1 magnification image obtained using the proposed imaging setup, we configured the imaging setup vertically to mimic the stereomicroscope orientation and used a mini-LED light array (Aputure Amaran AL-MX Bicolor LED) as a transmitted light source behind the resolution target. The raw photos taken by the imaging setup were shot at f/3.2 (optimum optical resolution), 1/40 s, and ISO 100, with the native resolution of 8688 × 5792 pixels.

We calculated the contrast from the difference in the amount of light in the greyscale (0 to 255) peaks “max” and valleys “min” between dark and bright line-pair patterns categorized from 0 to 100%. We used line probes in Avizo 2020.1 (Thermo Scientific, USA) to examine the intensity values of the target images quantitatively because this module scans the greyscale intensity values along a line probe assigned by the user. We identified the peaks and valleys that can be smoothed using sampling and averaging factors for better comparison. A graph of the contrast versus spatial frequency from both imaging systems can be obtained from the local modulation [48], given by Eq. (1):

1 $$\backslash (modulation=\backslash frac\left\{max-min\right\} \left\{max+min\right\} \backslash )$$

#### Root profilometry

We exported TIFF files containing the depth map layers from the stacks using Helicon Focus (Helicon Soft Ltd. v.7.5.6, Helicon Soft Ltd), Kharkov, Ukraine). These layers were processed with Avizo, starting with the determination of the scale using the known physical size of the images in *x*, *y*, and *z* (2.55 × 3.88 × 0.01 mm), where the micro-step determines *z* between layers within the stack. After loading the scaled dataset into Avizo, an image conversion step extracts the alpha channel from the stack made from the individual TIFF files. This channel is binarized to generate a tetrahedral mesh to reveal a triangulated surface of the root topography, which is carried out using the surface generation module in Avizo. Cleaning up the root-labeled channel may be necessary to obtain a result restricted to the region of interest because certain areas that do not represent the roots may be picked up due to uneven surfaces on the substrate where the root is growing or from dust accumulation in the sensor, creating ‘dust trails’ in the stack. Therefore, we advise to manually remove unwanted features in the Segmentation Editor in Avizo to apply a certain level of noise-smoothing and correct the contour roughness due to voxel aliasing, so that accurate and somewhat complex surfaces can be rendered with ease in Avizo. When necessary, the cleanup step should be performed before generating a mesh.

To visualize the 4D dynamics on the roots during dehydration and growth we configured the imaging setup as shown in Additional file 1: Fig. S6F, and we set the acquisition time to observe these dynamics in space and time. For example, during the dehydration observations (Fig. 4J), we captured three consecutive stacks (control/ hydrated, dehydrated, and rehydrated) made from 70 snaps at a 10-μm step in *z*, which generated 700-μm stacks imaged within 15 min. Therefore, the dynamics in a 700 μm stack imaged at a 10-μm resolution in *z* could not develop faster than the acquisition time (<15 min) to generate artifact-free datasets that could arise from specimen movement during acquisition. The image acquisition period was primarily bottlenecked by the Speedlight flashes that could not fire successively at full power, requiring a 12-s recharging delay between shots. Downtime could be improved by adding more light sources at lower relative power to allow for faster recharging.

#### High-throughput analysis of developmental, cell-cycle, and auxin mutants

We used the Multiple**XL**ab to examine root-growth dynamics in several different Arabidopsis mutants. Small Petri dishes were used to plate either 64 or 56 seeds on 1/2 MS agar mixed with charcoal to make the media dark, increasing the contrast between the roots and background. Plates were stored in a growth room (20.5 °C; 67% RH) for 24 h prior to being loaded into the carousel for hourly imaging for up to five days. The lid of the plates was removed to avoid condensation from blocking the view, and the entire device was enclosed using a plexiglass frame to minimize the drying of the agar plates and decelerate contamination that may happen after a week of ongoing experimentation.

### Statistical analysis

Data analysis was performed using computer software (OriginPro 2020, OriginLab, Massachusetts, USA). Datasets were significantly drawn from normally distributed populations according to the Shapiro–Wilk test using a 0.05 significance level [49]. The one-way analysis of variance followed by the post hoc Tukey test was employed using *p* < .05 to .001 to compare the difference between mutants.

### SeedNet and RootNet models

To determine the initial seed locations in the first frame of the time series, we developed an artificial neural network called SeedNet. The network is a binary image segmentation model based on the U-Net architecture [50]. Given an RGB image, SeedNet outputs a pixel-wise mask classifying each pixel as a seed [1] or not [0]. Raw images were too large to be processed with SeedNet; therefore, we divided the original image into patches of 256 × 256 pixels. Furthermore, we down sampled the patches to 32 × 32 pixels because we aimed to locate only the seed positions. SeedNet outputs were then upsampled to 256 × 256 pixel patches and stitched back together to obtain a pixel-wise mask with the original size of the raw image.

Similarly, we developed an artificial neural network called RootNet based on the same U‑Net architecture. RootNet outputs a pixel-wise mask classifying each pixel as a root [1] or not [0] from a given RGB image. Raw images were also too large for RootNet; therefore, we also patched (256 × 256 pixels) the original image. RootNet outputs were stitched back together to obtain a pixel-wise mask of the original-size raw image, like in the SeedNet outputs.

SeedNet and RootNet were developed using Keras [51], an open-source software library. The implementation details for the models are provided in the supplementary information (Additional file 1: Tables S2 and 3).

To train both RootNet and SeedNet models the dataset was randomly split into training [46 images] and testing [19 images] sets. Annotations were performed by labeling the root and seed pixels (Additional file 1: Fig. S7, B and D) using iLastik [52]. Each image had 64 seed/roots, hence a total of 3200 annotated plants. Since one entire image is too large to be used for training, each image was divided into smaller patches (256 × 256 pixels) and these patches were used to train the models. Thousands of patches were used for training each model and it took <10 minutes on a desktop computer with 30 GB of RAM and an NVIDIA Quadro P4000 GPU.

### Computing resources for image processing

For inference, the entire pipeline processing took about one hour to analyze a single timeseries on a laptop (8 GB RAM, CPU 2.6 GHz Intel Core i5), alternatively, and it took 30 minutes using a workstation (128GB RAM, Intel Xeon Gold 6130 @ 2.10GHz; NVIDIA Quadro M2000 GPU). Image processing tasks using Photoshop were also carried out using the laptop and workstation.

## Supplementary Information

**Additional file 1.** Additional figures and tables.

**Additional file 2: Movie S1.** Insects feeding on Arabidopsis flowers.

**Additional file 3: Movie S2.** Germination process and lateral roots.

**Additional file 4: Movie S3.** Soil organisms.

**Additional file 5: Movie S4.** Thrips feeding-off tomato’s root.

**Additional file 6: Movie S5.** Regeneration process in tomatoes.

**Additional file 7: Movie S6.** Root profilometry in 4D.

**Additional file 8: Movie S7.** Hybrid imaging set-up assembly tutorial.

**Additional file 9: Movie S8.** WT, *aux1*, and *lax3aux1* root growth.

**Additional file 10: Movie S9.** WT from auxin mutants group.

## Data Availability

All data are available in the main text or the supplementary materials.

## Footnotes

1 https://plantphenotyping.com/products/; https://www.qubitphenomics.com/products/.

2 https://plantphenotyping.com/products/plantscreen-root-system/.

3 https://www.qubitphenomics.com/rhizotube/.

4 https://www.phenospex.com/.

5 https://perma.cc/NW7W-6D6F.
