# Supplementary material for: MultipleXLab: A high-throughput portable live-imaging root phenotyping platform using deep learning and computer vision
Source: Plant Methods. 2022 Mar 27;18:38. doi: 10.1186/s13007-022-00864-4 (PMC8958799; doi:10.1186/s13007-022-00864-4)
Supplement: Supplementary file 1 — Additional file 1. Additional figures and tables. [file 13007_2022_864_MOESM1_ESM.docx]

Supplementary Materials

MultipleXLab: A high-throughput portable live-imaging root phenotyping platform using deep learning and computer vision

**Vinicius Lube, Mehmet Alican Noyan, Alexander Przybysz, Khaled Salama, Ikram Blilou***

*Corresponding author. Email: Ikram.Blilou@kaust,edu.sa

**The supplementary material file includes:**

Figs. S1 to S10

Tables S1 to S3

Movies S1 to S9


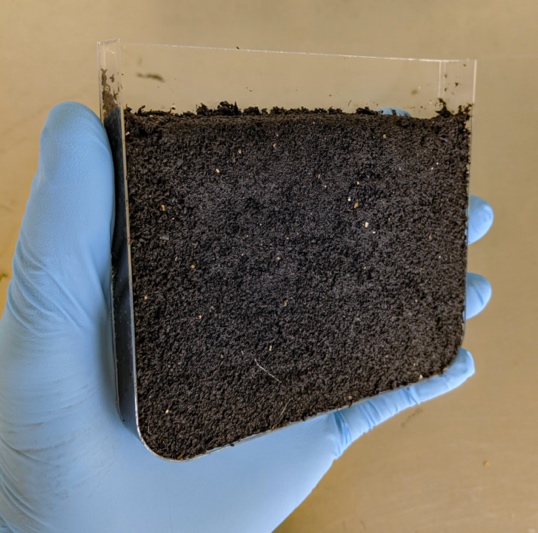

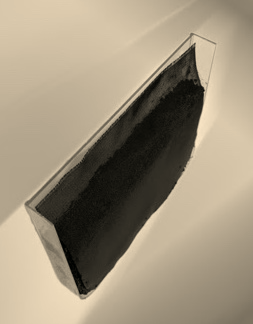

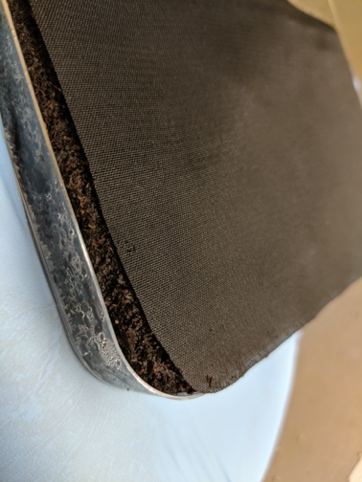


**B**

**A**

**C**

**D**

**E**

**F**


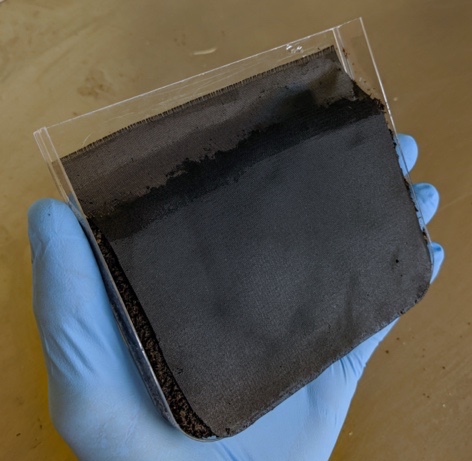

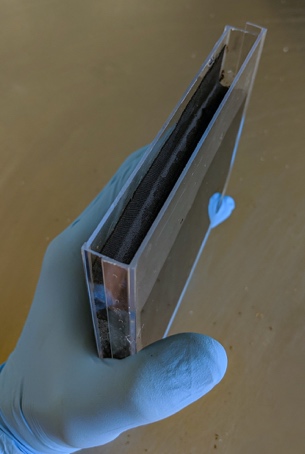

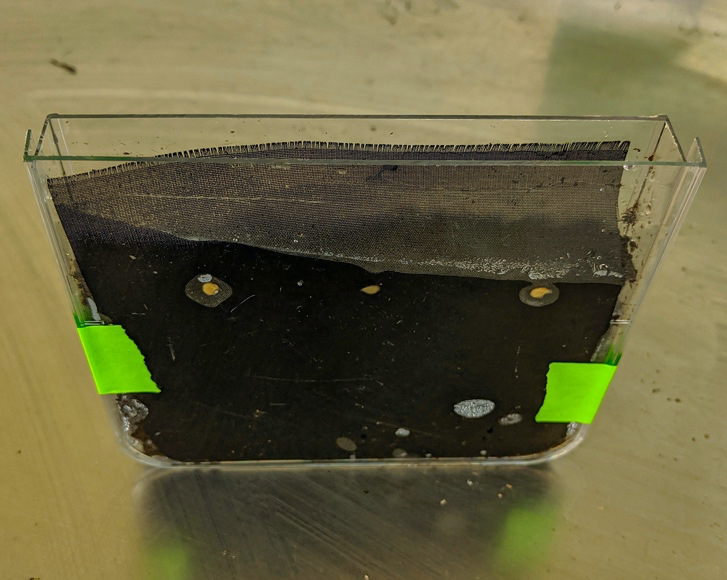


**Fig. S1** Mini-rhizotron setup. Made from a standard transparent polystyrene square Petri dish open at the top, with dimensions 12.5x10x5 cm. In (**A**) the mini-rhizotron was filled with soil, and then covered with the dark nylon mesh in (**B**), (**C**), and (**D**). In (**E**), the mini-rhizotron was closed using the acrylic lid and tape, and in (**F**), the final rhizotron is depicted with three tomato seeds plated on ¾ height on the mesh.


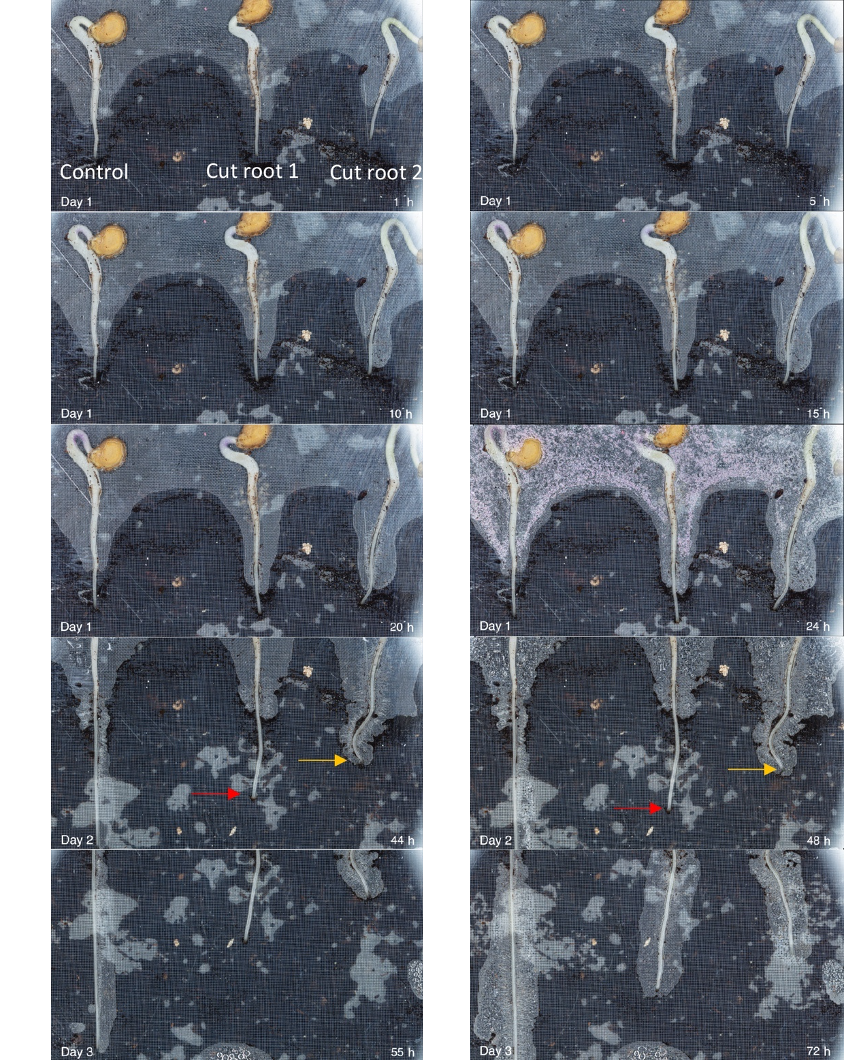


**J**

**I**

**H**

**G**

**E**

**F**

**D**

**B**

**A**

**C**

**Fig. S2** Regeneration studies using tomato roots. Root growth was followed after 1h (**A**). 5h (**B**), 10h (**C**), 15h (**D**), 20h (**E**), 24h (**F**), 44 (**G**), 48h (**H**), 55h (**I**), 72h (**J**). Regeneration took place after 45 to 50 hours in cut roots (**H**). Regeneration completed after 48 hours. (**I**) Normal growth rates resumed after 10 hours post-regeneration on the third day. (**J**) Final third-day mark of the fully regenerated roots. The field of view is 24x36 mm in all snaps. *N=3*. *N* is the number of tomato roots used for this experiments.

**
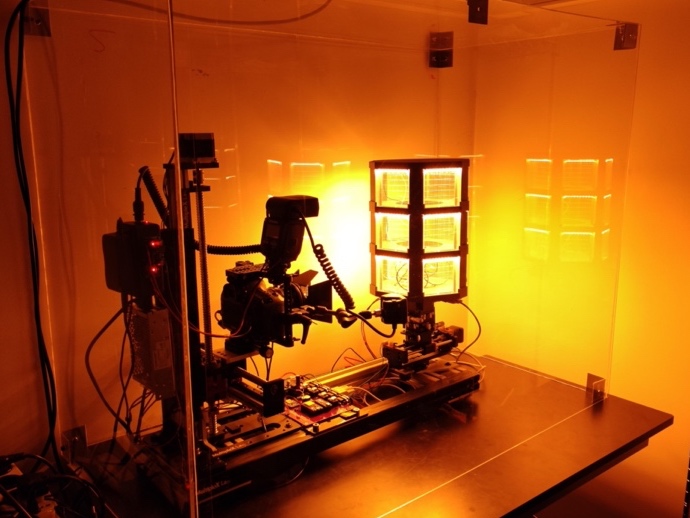
**
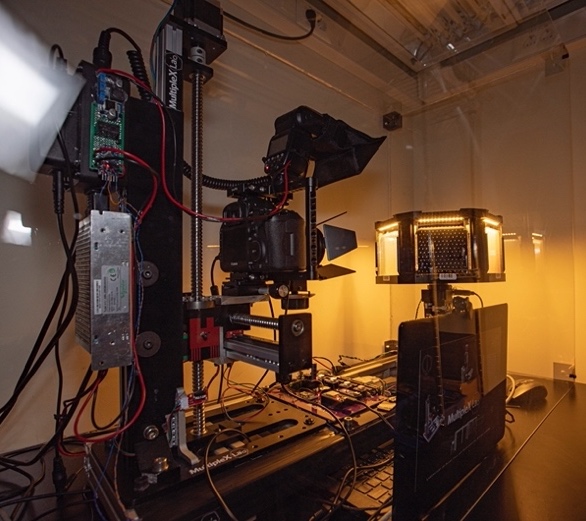


**B**

**A**

**C**

**D**

**
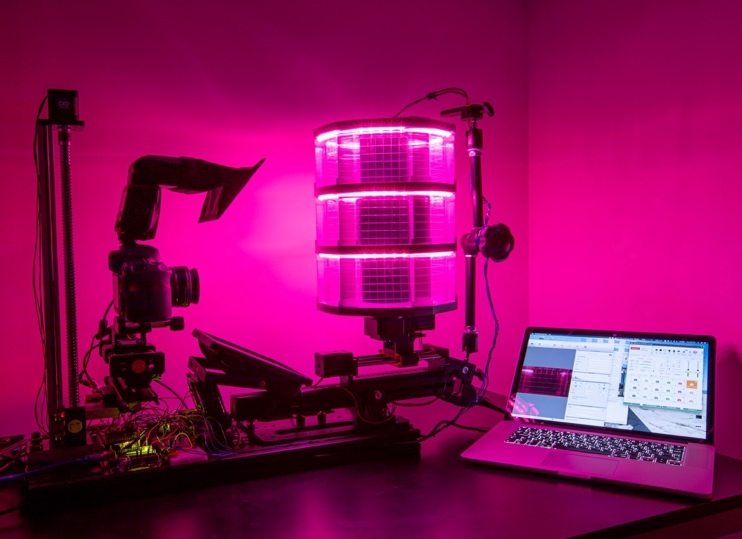
**
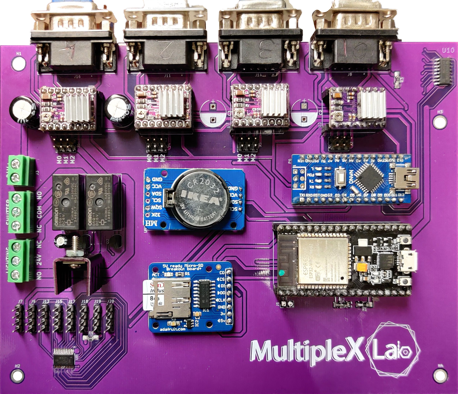


**E**


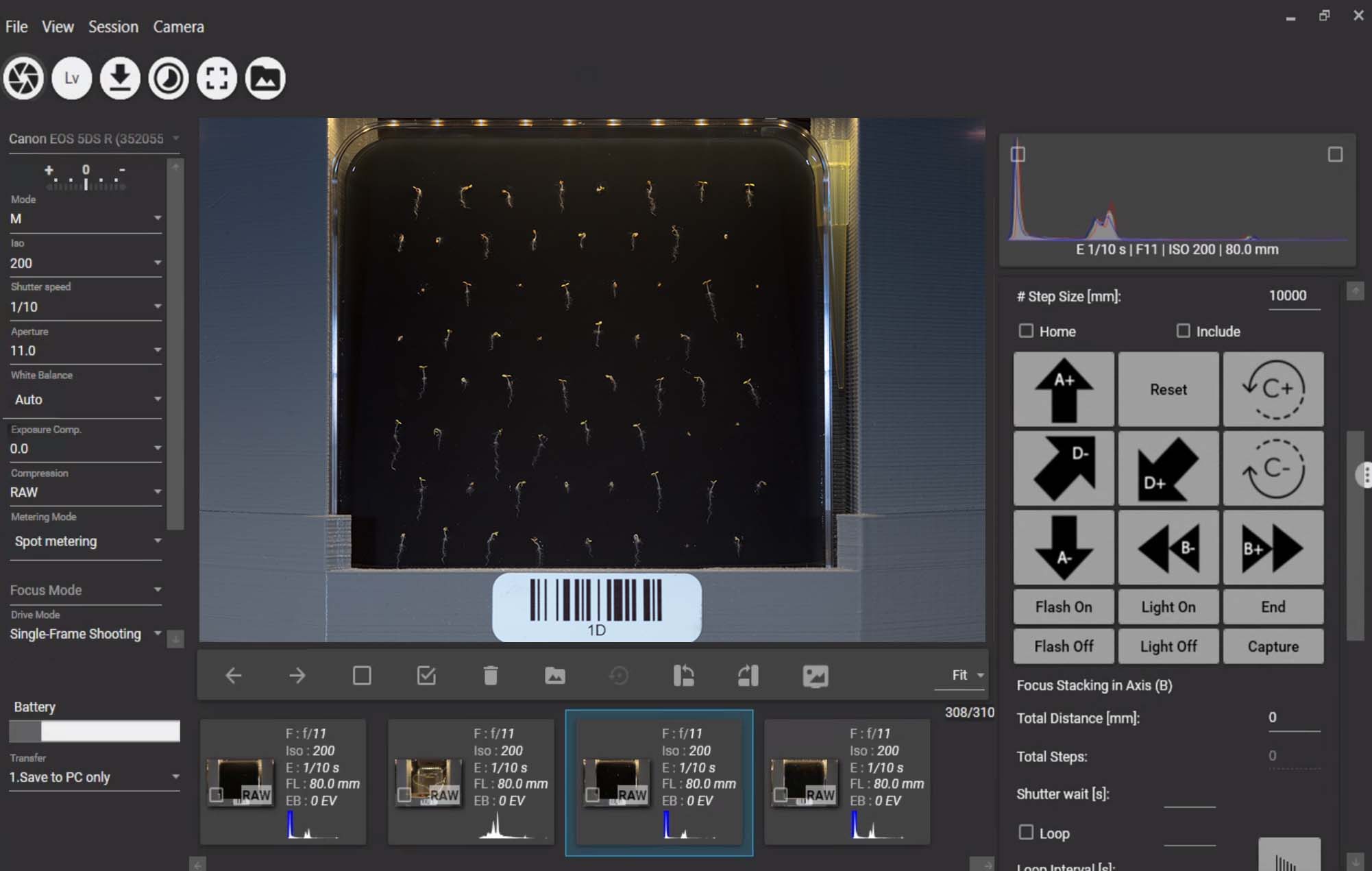


**Fig. S3** The Multiple**XL**ab device. (**A**) the system with the addition of a stage with 3 carousel levels is able to image 18 plates simultaneously. (**B**) stage for larger plates (120x120x16 mm) can also be used to accommodate larges specimens. (**C**) the system can operate with either plant-growth light or with white lighting in (**A**). (**D**) custom printed circuit board (PCB) uses an ESP32 as the master microcontroller to control the lighting and the camera shutter. It also reads a program from an SD card and logs any errors. Additionally, the ESP32 interfaces with an I2C port expander which enables controlling/communicating with auxiliary sensors/actuators, as well as a real-time clock (RTC) module to synchronize the program timings and the light cycles accurately. The Arduino Nano is a slave that acts as a liaison to the stepper motors. It receives commands from the ESP32 and performs the stepper operations. The Arduino Nano also handles limit switches used in the calibration step. (**E**) Multiple**XL**ab Control Center software UI used to control the lighting and acquire images, as well as to perform calibration and focus stacking.


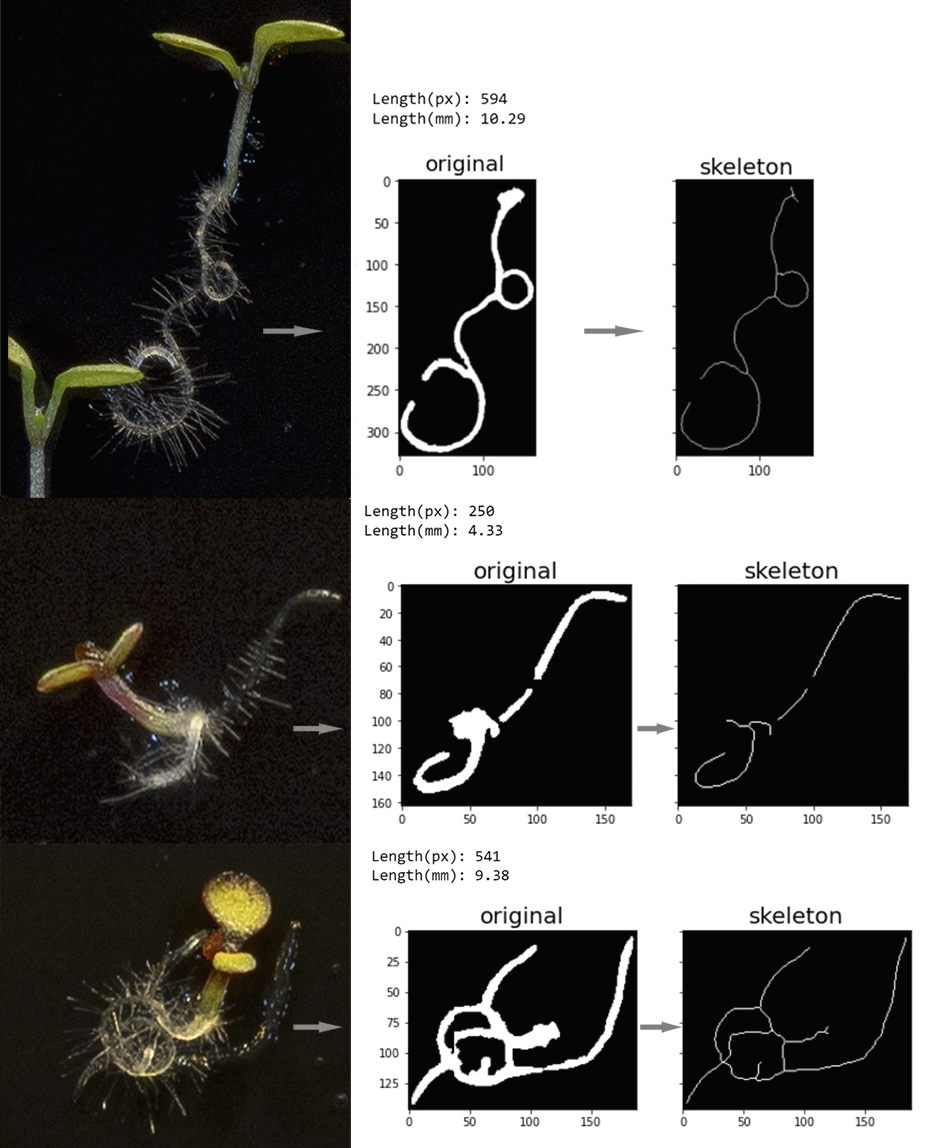


**D**

**C**

**B**

**A**


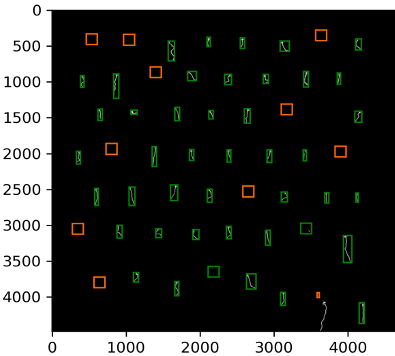


**Fig. S4** Agravitropic auxin transport mutants (*aux1)* after 4 days of growth. (**A**) curling of the root caused it to obstruct itself in certain parts of the root and neighboring cotyledons (red arrow), therefore introducing small errors in the root length measurements. (**B**) root discontinuity due to lack of contrast as a result of the agar substrate slightly covering the root (red arrow) in that short segment. (**C**) example image showing the usefulness of the time-lapse imaging combined with computer vision techniques to optimize the mapping of the root path upon root growth, whereas a person would encounter difficulties attempting to skeletonize this root path manually without precedent timepoints in this example. Graph scales are represented in pixels with a reference to mm on the sub-figures. (**D**) example of detection and tracking failure in one seed on the bottom row. Scale in (**D**) in pixels (57.3 pixels/mm).


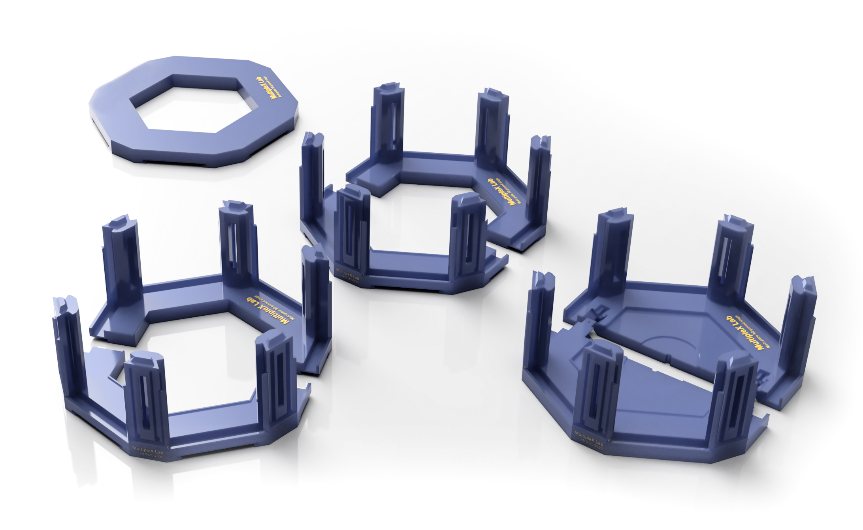

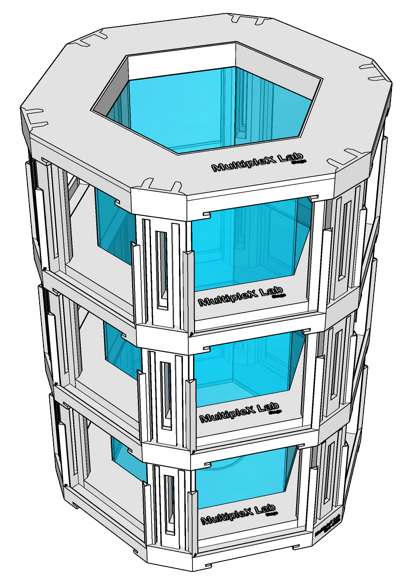


**D**

**C**

**A**

**B**


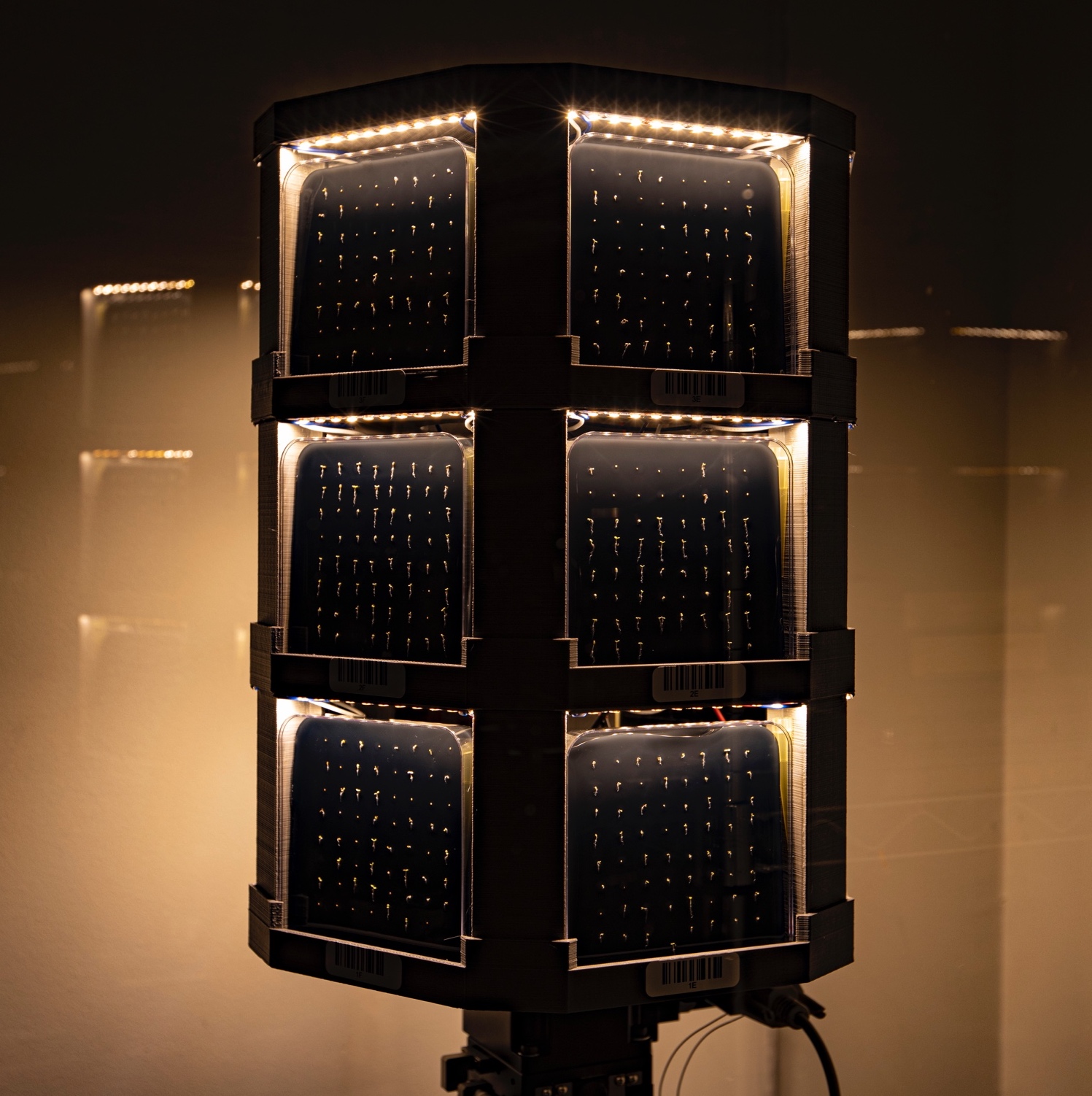

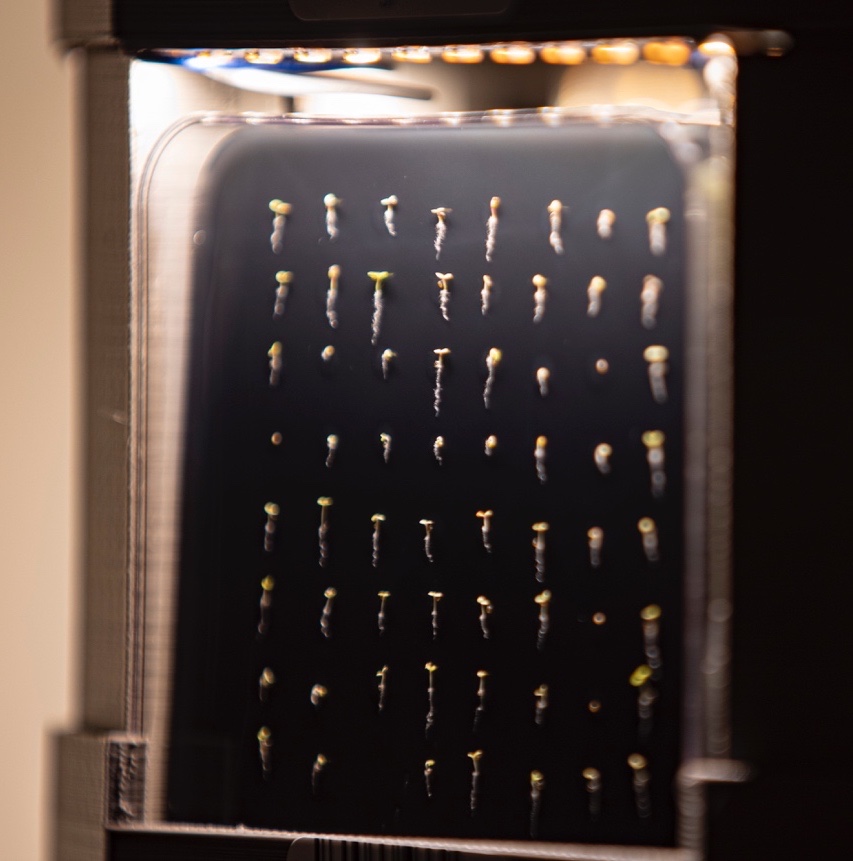


**Fig. S5** Carousel 3D design. (**A**) CGI of the larger carousel used with the larger Petri dish plates (120x120x16 mm) depicting the modularity of the design; the pillars and the hexagonal carousel are retractable to increase portability during transportation. (**B**) CGI of the stacked multi-plate 3D design of the carousel. (**C**) real image of the carrousel loaded with 18 plates (1152 seeds), with a close-up of a plate in (**D**).


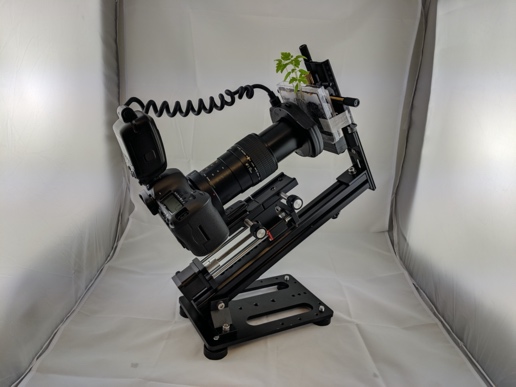

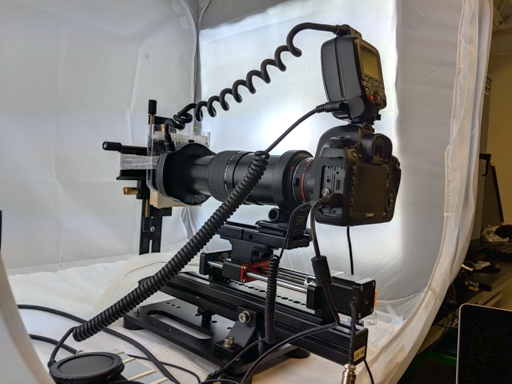

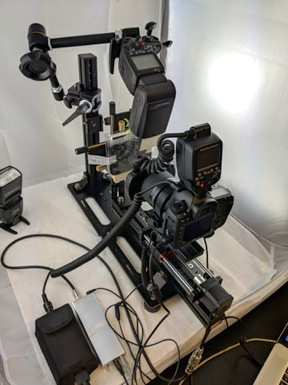


**C**

**F**

**I**

**E**

**D**

**G**

**B**

**A**


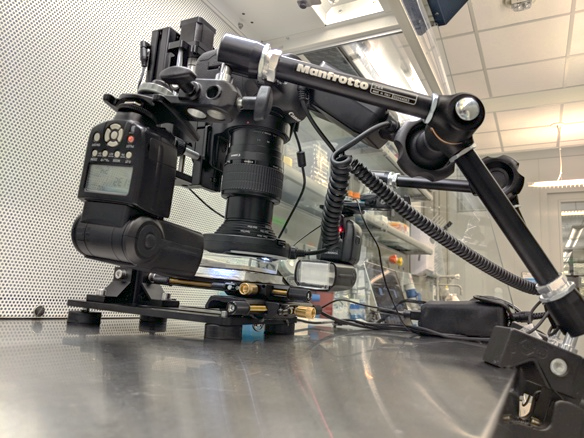

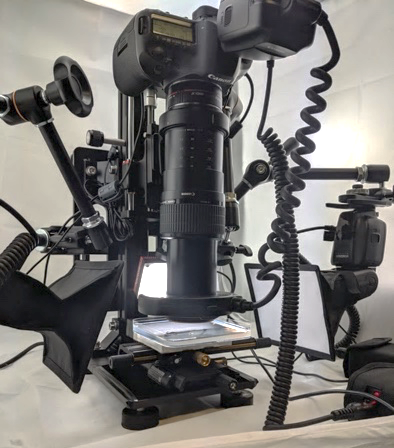

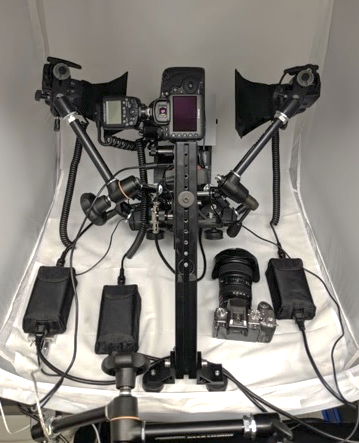


**H**


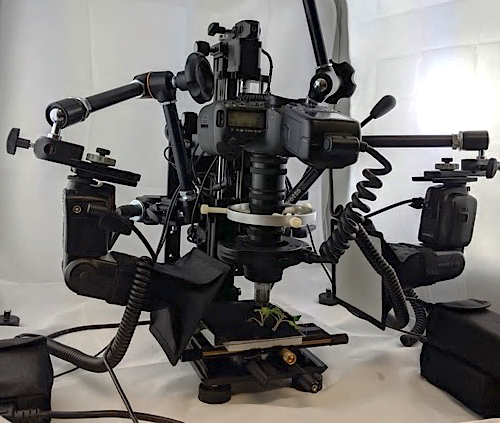

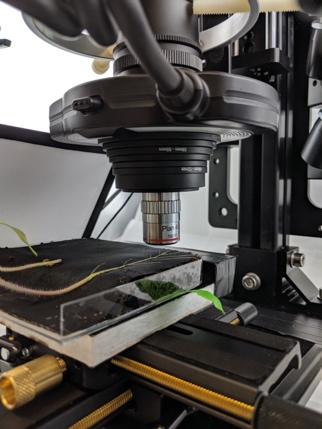

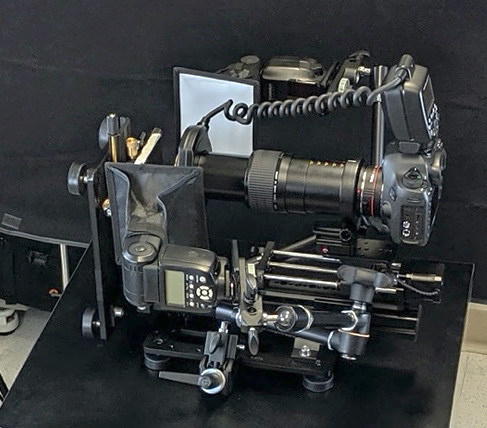


**Fig. S6** Imaging setup depicted in several different configurations as per end-user requirements. (**A**) Simple configuration on the imaging setup at 45° inclination. (**B**-**C**) Horizontal configurations using one oblique ring flash (**B**) and also one Speedlite flash (**C**) on top. (**D**-**E**) Vertical orientation with oblique and lateral flashes in the laminar flow hood (**D**) and in the lightbox (**E**). (**F**) depicts the -45° inclination used during the dehydration studies. (**G**-**H**) Microscope objectives were easily mounted on the imaging setup; (**H**) depicts a close-up image of (**G**). (**I**) Hybrid configuration of the imaging setup allowing it to be easily alternated from horizontal to vertical orientations simply by turning the entire setup because it has two bases in that configuration, as well as all the lighting systems in a fixed position.


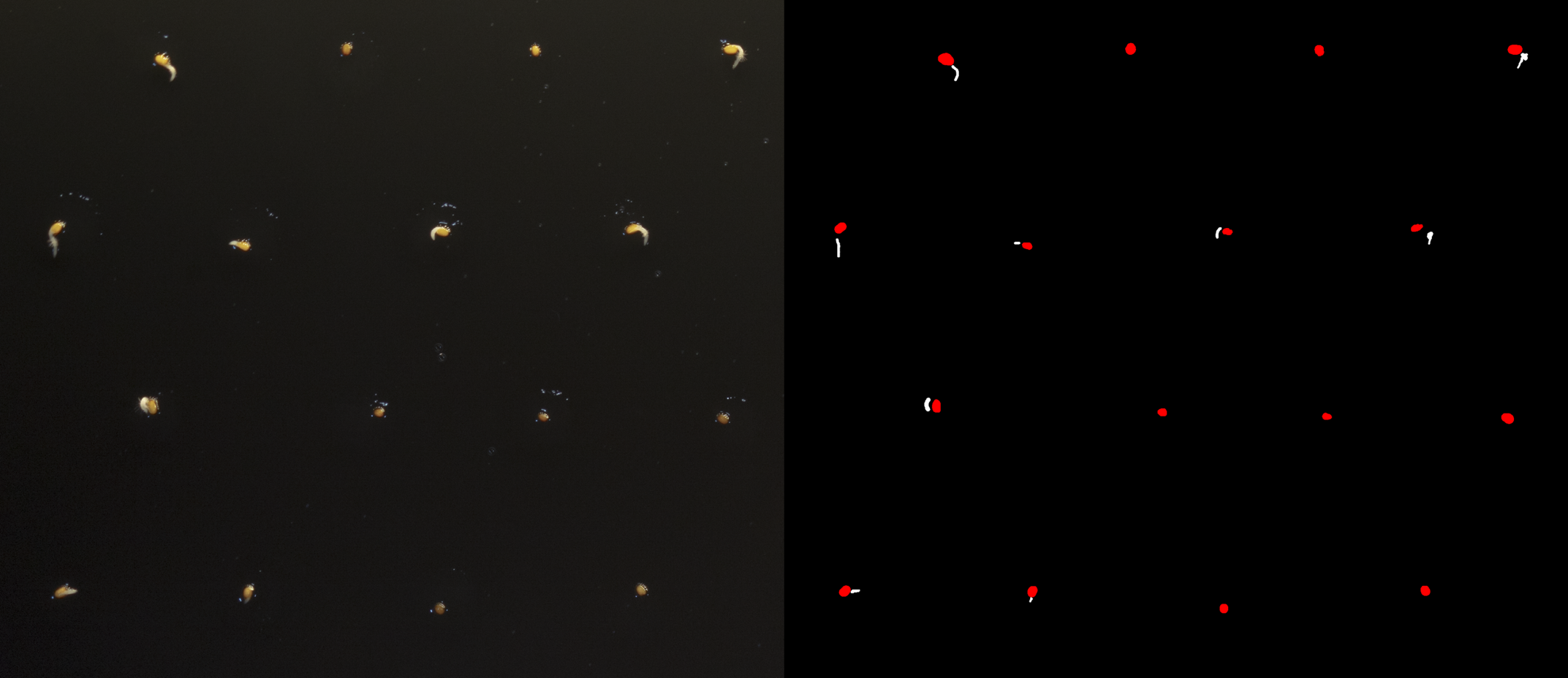


**A**

**B**


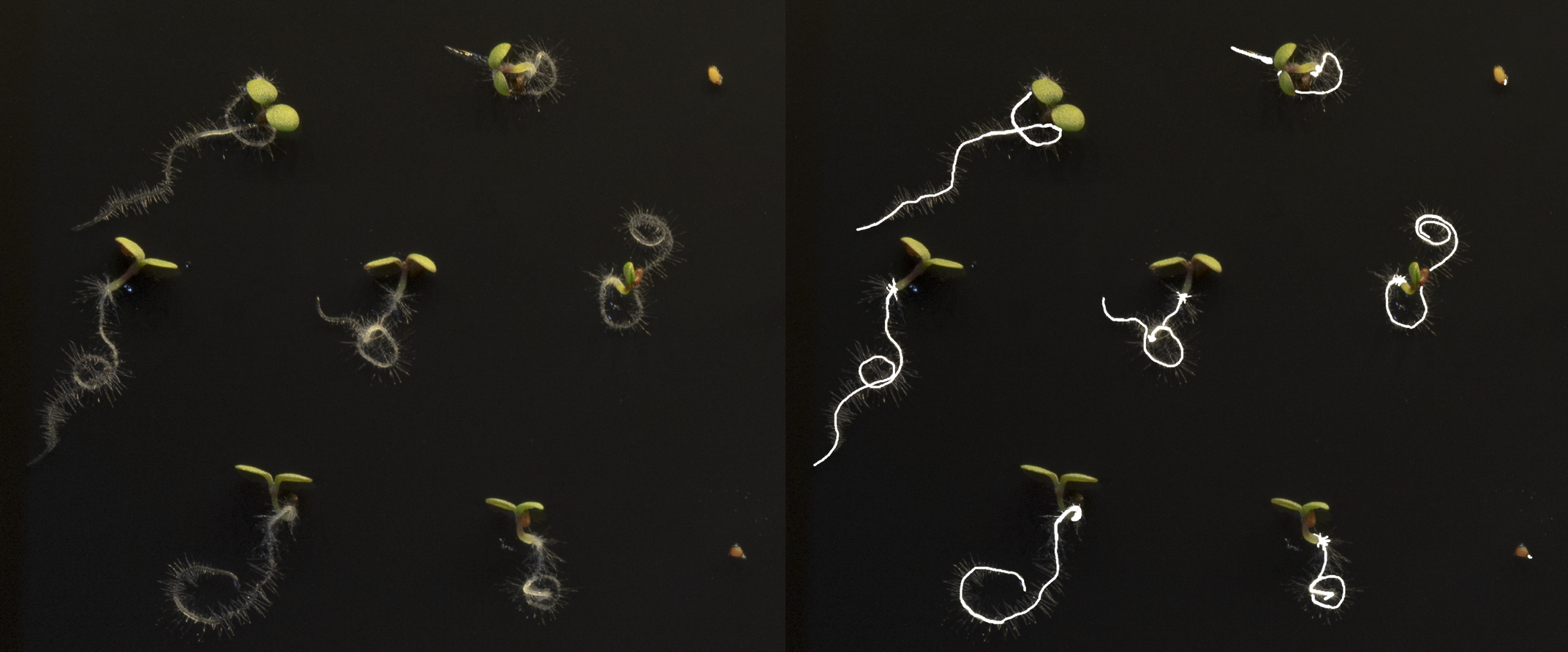


**D**

**C**

**Fig. S7** Manually annotated images of auxin mutants used for training SeedNet and RootNet. (**A**) and (**B**) are the same timepoint whereas the seed coat label is overlaid in (**B**) in red, and the root label is represented in white. (**C**) and (**D**) also belong to the same timepoint, the root label is overlaid in (**D**) in white. Scale bar is 1 cm.


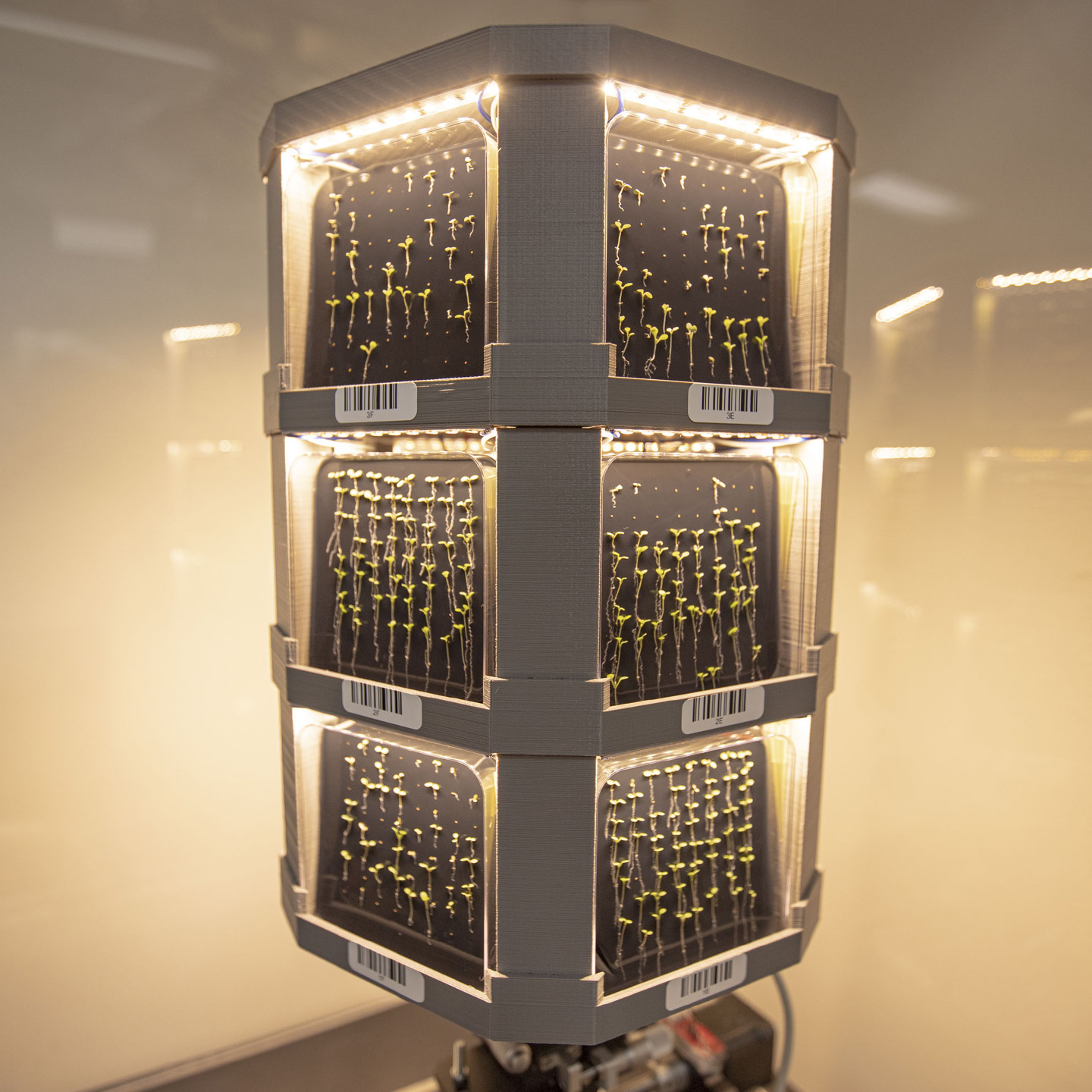


**Fig. S8** EMS mutagenesis screen to isolate mutants with germination phenotypes after 6 days of growth. Scale bar is 2 cm.

**
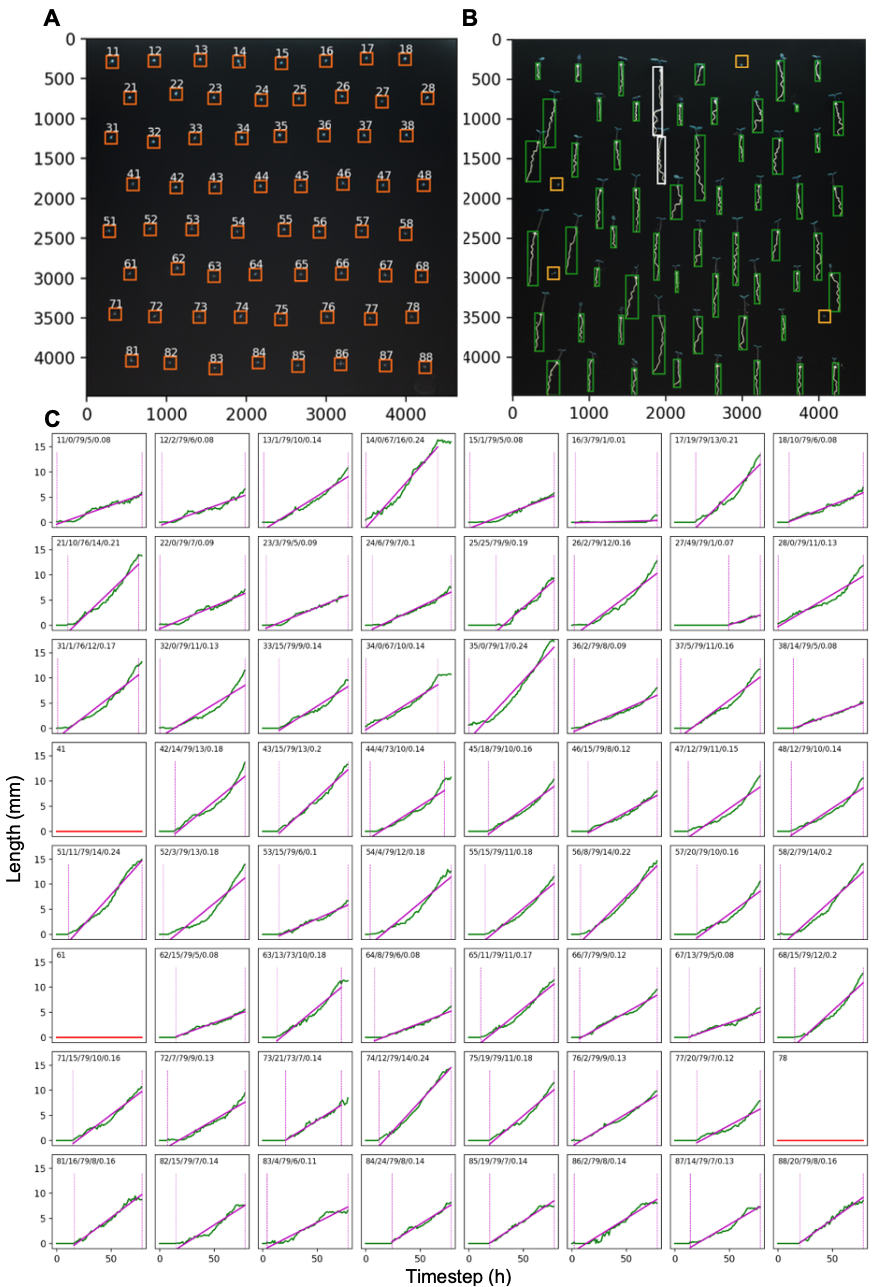
**

**Fig. S9** Germination dashboard showing complete plots of individual Wild-type (WT) root lengths from germination to the last viable timepoint for measurement. The group of five numbers on top of the plots represent the i) seed/root ID (row, column), ii) timepoint in which germination began (in hours), iii) timepoint for the last measured timepoint (in hours), iv) root length (mm) at the last viable timepoint, and v) growth rate of roots (mm/h). The red horizontal lines in seed IDs 41, 61, and 78 indicate nongermination. This graph showing 64 specimens complements Fig. 6.

**
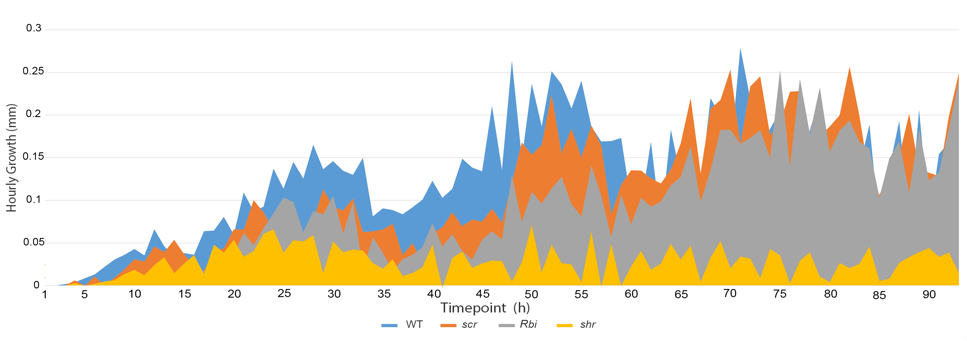
**

Hourly Growth (mm)

**B**

**A**

**
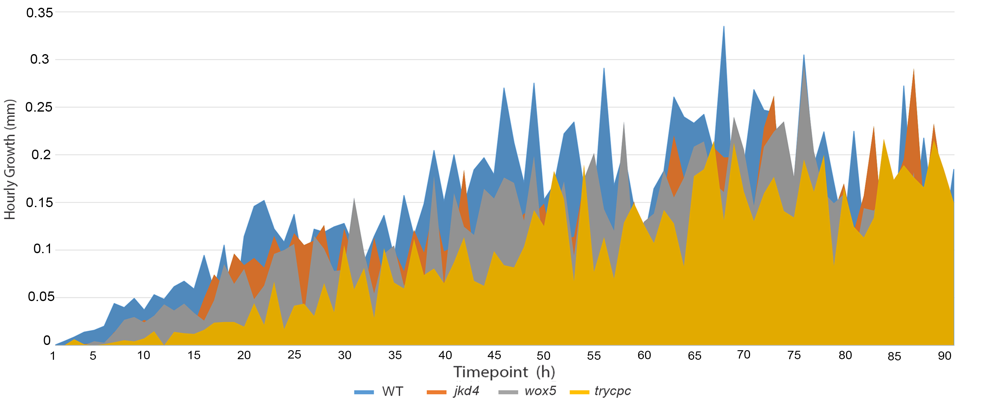
**

Hourly Growth (mm)

**C**

**
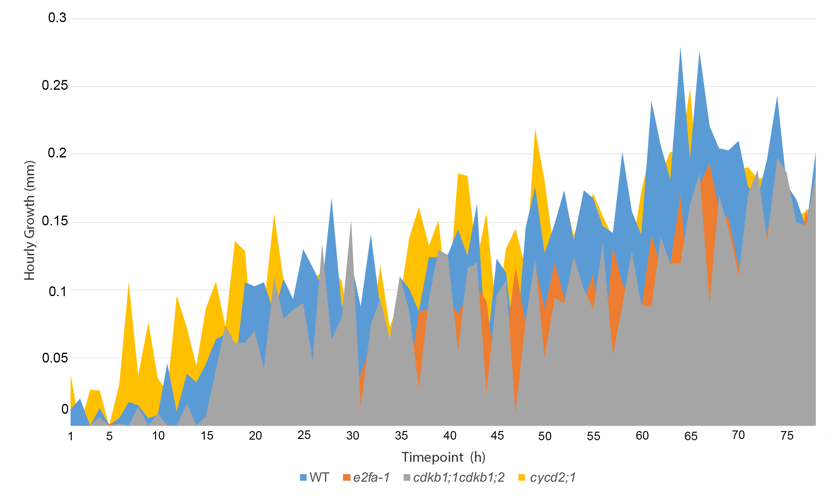
**

**D**

Hourly Growth (mm)

**
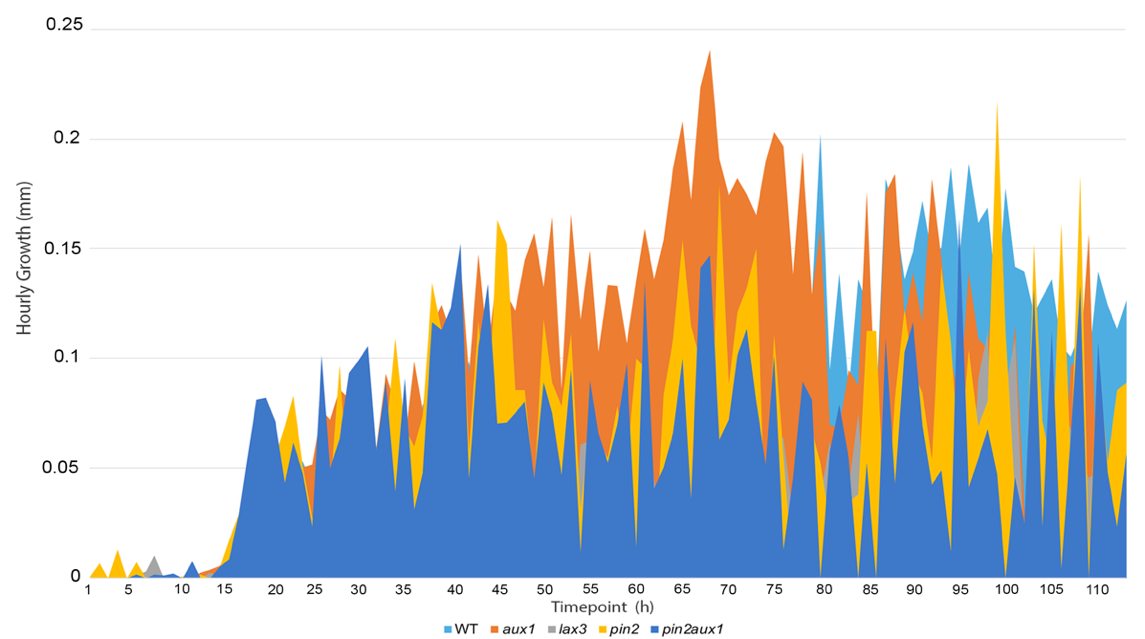
**

Hourly Growth (mm)

**Fig. S10** Graphs depicting the hourly growth throughout the growth cycle for all mutants analyzed in this study. (**A**) complements Fig. 7C, (**B**) complements Fig. 8C, (**C**) ­complements Fig. 9C, and (**D**) complements Fig. 10C. ­

**Table S1** Single-plate Imaging setup components and cost

| **Component** | **Brand/Model Number** | **Function** | **Cost (USD)** |
| --- | --- | --- | --- |
| DSLR full-spectrum camera | DSLR Camera Canon EOS 5DS R - 50.6 MP + full spectrum conversion | Long-term live-imaging device | 1,499.00 |
| Macro-lens | Macro-lens Canon MP-E 65mm f/2.8 1-5x Macro Photo | Lens capable of producing 5:1 life-size magnification | 1,049.00 |
| General purpose lens | Prime lens Canon 40mm EF 40mm f/2.8 STM | Normal-length prime lens used for the large field of view in high-throughput applications | 179.00 |
| Diopter filter | Kenko Close-Up Lens 58mm AC No.5 Achromatic-Lens | This filter shifts the minimal focus distance of the lens so that the camera can be closer to the specimen, thus increasing magnification | 45.38 |
| Teleconverter | Vivitar Series 1 Teleconverter lens for Canon (2:1 magnification) | Focal length extender used to increase magnification | 109.95 |
| Visible filter | External Mounted Visible Bandpass Filters (58 mm filter diameter) | Ultraviolet and Infrared blocking filter | 90 |
| Macro-rail step-motor | WeMacro Rail (100mm) | Focus bracketing | 249 |
| Main camera stage | WeMacro Vertical and Horizontal Stand (2.0) | Used to place the camera/sample stage vertically or horizontally. An additional unit can be purchased to make the set-up horizontally/vertically hybrid to seamlessly allow conversion from vertical to horizontal orientation, which is accomplished by simply attaching another base plate parallel to the center column | 129.9 |
| Articulated arms | Two Manfrotto 244 Variable Friction Magic Arm with Camera Bracket with Manfrotto 035 Super Clamp without Stud | Hold flashes in any position | 318.94 |
| X-Y sample stage | Two Manfrotto 454 Micrometric Positioning Sliding Plate | X and Y micro-positioning of samples mounted on a base made from plywood covered with black velvet | 180 |
|  |  |  |  |
| Speedlight Flash #1 | Canon MR-14EX II Macro Ring Lite | Ring light responsible for producing key light directly onto the sample | 549 |
| Speedlight Flashes #2 and 3 | Two Yongnuo Speedlite YN600EX-RT II | These external flashes can be used as fill light in the lightbox. Alternatively, they can be used to generate oblique light onto the specimens | 226 |
| Memory card | Memory card SanDisk 128GB Extreme PRO UHS-I SDXC Memory Card (V30) | Memory card used to save images remotely and independently of a tethered computer | 34.99 |
| Lightbox | Photography Kit - 80x80cms Soft Box Tent 60x60cms Soft Lights (150W) | Provide a chamber with steady airflow & fill light used to place the camera stage and samples. It also allows capturing shadowless images of complex surfaces to a certain degree | 191.98 |
|  |  |  |  |
| Camera power supply | Canon AC-E6N AC Adapter and DC Coupler DR-E6 Kit | Studio power supply adapter for camera | 143.95 |
| Flash power supply | Tronix SpeedFire Power Supply by Innovatronix | Adapter that provides continue power supply to the external flash units | 135 |
| Remote battery supply | 12 AA batteries Panasonic Eneloop Pro AA High Capacity NiMH | Batteries used in the Speedlite flash units | 70 |
| Mini LED light array | Aputure Amaran AL-MX Bicolor LED | Provide soft white and warm light underneath samples such as glass slides and plates. Used to attain backlighting or brightfield illumination. | 149 |
| Helicon Focus | Stacking and 3D imaging | Allows to merge z-stacks to create focus staking and increase DOF | 55 |
| Computer | Any simple laptop running Windows or Mac OS | System control for image acquisition parameters and stacking | - |
| **Total** | | | **5,405.09** |

**Table S2** SeedNet

| **Optimizer** | **Loss** | **Batch size** | **Epoch** |
| --- | --- | --- | --- |
| Adam | Binary cross entropy | 16 | Early stopping with patience 7 |

Layer (type) Output Shape Param # Connected to

==================================================================================================

input_1 (InputLayer) [(None, 32, 32, 3)] 0

__________________________________________________________________________________________________

conv2d (Conv2D) (None, 32, 32, 16) 448 input_1[0][0]

__________________________________________________________________________________________________

dropout (Dropout) (None, 32, 32, 16) 0 conv2d[0][0]

__________________________________________________________________________________________________

conv2d_1 (Conv2D) (None, 32, 32, 16) 2320 dropout[0][0]

__________________________________________________________________________________________________

max_pooling2d (MaxPooling2D) (None, 16, 16, 16) 0 conv2d_1[0][0]

__________________________________________________________________________________________________

conv2d_2 (Conv2D) (None, 16, 16, 32) 4640 max_pooling2d[0][0]

__________________________________________________________________________________________________

dropout_1 (Dropout) (None, 16, 16, 32) 0 conv2d_2[0][0]

__________________________________________________________________________________________________

conv2d_3 (Conv2D) (None, 16, 16, 32) 9248 dropout_1[0][0]

__________________________________________________________________________________________________

max_pooling2d_1 (MaxPooling2D) (None, 8, 8, 32) 0 conv2d_3[0][0]

__________________________________________________________________________________________________

conv2d_4 (Conv2D) (None, 8, 8, 64) 18496 max_pooling2d_1[0][0]

__________________________________________________________________________________________________

dropout_2 (Dropout) (None, 8, 8, 64) 0 conv2d_4[0][0]

__________________________________________________________________________________________________

conv2d_5 (Conv2D) (None, 8, 8, 64) 36928 dropout_2[0][0]

__________________________________________________________________________________________________

max_pooling2d_2 (MaxPooling2D) (None, 4, 4, 64) 0 conv2d_5[0][0]

__________________________________________________________________________________________________

conv2d_6 (Conv2D) (None, 4, 4, 128) 73856 max_pooling2d_2[0][0]

__________________________________________________________________________________________________

dropout_3 (Dropout) (None, 4, 4, 128) 0 conv2d_6[0][0]

__________________________________________________________________________________________________

conv2d_7 (Conv2D) (None, 4, 4, 128) 147584 dropout_3[0][0]

__________________________________________________________________________________________________

max_pooling2d_3 (MaxPooling2D) (None, 2, 2, 128) 0 conv2d_7[0][0]

__________________________________________________________________________________________________

conv2d_8 (Conv2D) (None, 2, 2, 256) 295168 max_pooling2d_3[0][0]

__________________________________________________________________________________________________

dropout_4 (Dropout) (None, 2, 2, 256) 0 conv2d_8[0][0]

__________________________________________________________________________________________________

conv2d_9 (Conv2D) (None, 2, 2, 256) 590080 dropout_4[0][0]

__________________________________________________________________________________________________

conv2d_transpose (Conv2DTranspo (None, 4, 4, 128) 131200 conv2d_9[0][0]

__________________________________________________________________________________________________

concatenate (Concatenate) (None, 4, 4, 256) 0 conv2d_transpose[0][0]

conv2d_7[0][0]

__________________________________________________________________________________________________

conv2d_10 (Conv2D) (None, 4, 4, 128) 295040 concatenate[0][0]

__________________________________________________________________________________________________

dropout_5 (Dropout) (None, 4, 4, 128) 0 conv2d_10[0][0]

__________________________________________________________________________________________________

conv2d_11 (Conv2D) (None, 4, 4, 128) 147584 dropout_5[0][0]

__________________________________________________________________________________________________

conv2d_transpose_1 (Conv2DTrans (None, 8, 8, 64) 32832 conv2d_11[0][0]

__________________________________________________________________________________________________

concatenate_1 (Concatenate) (None, 8, 8, 128) 0 conv2d_transpose_1[0][0]

conv2d_5[0][0]

__________________________________________________________________________________________________

conv2d_12 (Conv2D) (None, 8, 8, 64) 73792 concatenate_1[0][0]

__________________________________________________________________________________________________

dropout_6 (Dropout) (None, 8, 8, 64) 0 conv2d_12[0][0]

__________________________________________________________________________________________________

conv2d_13 (Conv2D) (None, 8, 8, 64) 36928 dropout_6[0][0]

__________________________________________________________________________________________________

conv2d_transpose_2 (Conv2DTrans (None, 16, 16, 32) 8224 conv2d_13[0][0]

__________________________________________________________________________________________________

concatenate_2 (Concatenate) (None, 16, 16, 64) 0 conv2d_transpose_2[0][0]

conv2d_3[0][0]

__________________________________________________________________________________________________

conv2d_14 (Conv2D) (None, 16, 16, 32) 18464 concatenate_2[0][0]

__________________________________________________________________________________________________

dropout_7 (Dropout) (None, 16, 16, 32) 0 conv2d_14[0][0]

__________________________________________________________________________________________________

conv2d_15 (Conv2D) (None, 16, 16, 32) 9248 dropout_7[0][0]

__________________________________________________________________________________________________

conv2d_transpose_3 (Conv2DTrans (None, 32, 32, 16) 2064 conv2d_15[0][0]

__________________________________________________________________________________________________

concatenate_3 (Concatenate) (None, 32, 32, 32) 0 conv2d_transpose_3[0][0]

conv2d_1[0][0]

__________________________________________________________________________________________________

conv2d_16 (Conv2D) (None, 32, 32, 16) 4624 concatenate_3[0][0]

__________________________________________________________________________________________________

dropout_8 (Dropout) (None, 32, 32, 16) 0 conv2d_16[0][0]

__________________________________________________________________________________________________

conv2d_17 (Conv2D) (None, 32, 32, 16) 2320 dropout_8[0][0]

__________________________________________________________________________________________________

conv2d_18 (Conv2D) (None, 32, 32, 1) 17 conv2d_17[0][0]

==================================================================================================

Total params: 1,941,105

Trainable params: 1,941,105

Non-trainable params: 0

__________________________________________________________________________________________________

**Table S3** RootNet

| **Optimizer** | **Loss** | **Batch size** | **Epoch** |
| --- | --- | --- | --- |
| Adam | Binary cross entropy | 16 | Early stopping with patience 7 |

Layer (type) Output Shape Param # Connected to

==================================================================================================

input_1 (InputLayer) [(None, 256, 256, 3) 0

__________________________________________________________________________________________________

conv2d (Conv2D) (None, 256, 256, 16) 448 input_1[0][0]

__________________________________________________________________________________________________

dropout (Dropout) (None, 256, 256, 16) 0 conv2d[0][0]

__________________________________________________________________________________________________

conv2d_1 (Conv2D) (None, 256, 256, 16) 2320 dropout[0][0]

__________________________________________________________________________________________________

max_pooling2d (MaxPooling2D) (None, 128, 128, 16) 0 conv2d_1[0][0]

__________________________________________________________________________________________________

conv2d_2 (Conv2D) (None, 128, 128, 32) 4640 max_pooling2d[0][0]

__________________________________________________________________________________________________

dropout_1 (Dropout) (None, 128, 128, 32) 0 conv2d_2[0][0]

__________________________________________________________________________________________________

conv2d_3 (Conv2D) (None, 128, 128, 32) 9248 dropout_1[0][0]

__________________________________________________________________________________________________

max_pooling2d_1 (MaxPooling2D) (None, 64, 64, 32) 0 conv2d_3[0][0]

__________________________________________________________________________________________________

conv2d_4 (Conv2D) (None, 64, 64, 64) 18496 max_pooling2d_1[0][0]

__________________________________________________________________________________________________

dropout_2 (Dropout) (None, 64, 64, 64) 0 conv2d_4[0][0]

__________________________________________________________________________________________________

conv2d_5 (Conv2D) (None, 64, 64, 64) 36928 dropout_2[0][0]

__________________________________________________________________________________________________

max_pooling2d_2 (MaxPooling2D) (None, 32, 32, 64) 0 conv2d_5[0][0]

__________________________________________________________________________________________________

conv2d_6 (Conv2D) (None, 32, 32, 128) 73856 max_pooling2d_2[0][0]

__________________________________________________________________________________________________

dropout_3 (Dropout) (None, 32, 32, 128) 0 conv2d_6[0][0]

__________________________________________________________________________________________________

conv2d_7 (Conv2D) (None, 32, 32, 128) 147584 dropout_3[0][0]

__________________________________________________________________________________________________

max_pooling2d_3 (MaxPooling2D) (None, 16, 16, 128) 0 conv2d_7[0][0]

__________________________________________________________________________________________________

conv2d_8 (Conv2D) (None, 16, 16, 256) 295168 max_pooling2d_3[0][0]

__________________________________________________________________________________________________

dropout_4 (Dropout) (None, 16, 16, 256) 0 conv2d_8[0][0]

__________________________________________________________________________________________________

conv2d_9 (Conv2D) (None, 16, 16, 256) 590080 dropout_4[0][0]

__________________________________________________________________________________________________

conv2d_transpose (Conv2DTranspo (None, 32, 32, 128) 131200 conv2d_9[0][0]

__________________________________________________________________________________________________

concatenate (Concatenate) (None, 32, 32, 256) 0 conv2d_transpose[0][0]

conv2d_7[0][0]

__________________________________________________________________________________________________

conv2d_10 (Conv2D) (None, 32, 32, 128) 295040 concatenate[0][0]

__________________________________________________________________________________________________

dropout_5 (Dropout) (None, 32, 32, 128) 0 conv2d_10[0][0]

__________________________________________________________________________________________________

conv2d_11 (Conv2D) (None, 32, 32, 128) 147584 dropout_5[0][0]

__________________________________________________________________________________________________

conv2d_transpose_1 (Conv2DTrans (None, 64, 64, 64) 32832 conv2d_11[0][0]

__________________________________________________________________________________________________

concatenate_1 (Concatenate) (None, 64, 64, 128) 0 conv2d_transpose_1[0][0]

conv2d_5[0][0]

__________________________________________________________________________________________________

conv2d_12 (Conv2D) (None, 64, 64, 64) 73792 concatenate_1[0][0]

__________________________________________________________________________________________________

dropout_6 (Dropout) (None, 64, 64, 64) 0 conv2d_12[0][0]

__________________________________________________________________________________________________

conv2d_13 (Conv2D) (None, 64, 64, 64) 36928 dropout_6[0][0]

__________________________________________________________________________________________________

conv2d_transpose_2 (Conv2DTrans (None, 128, 128, 32) 8224 conv2d_13[0][0]

__________________________________________________________________________________________________

concatenate_2 (Concatenate) (None, 128, 128, 64) 0 conv2d_transpose_2[0][0]

conv2d_3[0][0]

__________________________________________________________________________________________________

conv2d_14 (Conv2D) (None, 128, 128, 32) 18464 concatenate_2[0][0]

__________________________________________________________________________________________________

dropout_7 (Dropout) (None, 128, 128, 32) 0 conv2d_14[0][0]

__________________________________________________________________________________________________

conv2d_15 (Conv2D) (None, 128, 128, 32) 9248 dropout_7[0][0]

__________________________________________________________________________________________________

conv2d_transpose_3 (Conv2DTrans (None, 256, 256, 16) 2064 conv2d_15[0][0]

__________________________________________________________________________________________________

concatenate_3 (Concatenate) (None, 256, 256, 32) 0 conv2d_transpose_3[0][0]

conv2d_1[0][0]

__________________________________________________________________________________________________

conv2d_16 (Conv2D) (None, 256, 256, 16) 4624 concatenate_3[0][0]

__________________________________________________________________________________________________

dropout_8 (Dropout) (None, 256, 256, 16) 0 conv2d_16[0][0]

__________________________________________________________________________________________________

conv2d_17 (Conv2D) (None, 256, 256, 16) 2320 dropout_8[0][0]

__________________________________________________________________________________________________

conv2d_18 (Conv2D) (None, 256, 256, 1) 17 conv2d_17[0][0]

==================================================================================================

Total params: 1,941,105

Trainable params: 1,941,105

Non-trainable params: 0

__________________________________________________________________________________________________

**Movie S1 - Insects feeding on Arabidopsis flowers.**

**Movie S2 - Germination process and lateral roots.**

**Movie S3 - Soil organisms live imaging.**

**Movie S4 - Thrips feeding-off tomato’s root.**

**Movie S5 - Regeneration process in tomatoes.**

**Movie S6 - Root profilometry in 4D.**

**Movie S7 - Hybrid imaging set-up assembly (tutorial).**

**Movie S8 - WT, aux1, and lax3aux1 root growth.**

**Movie S9 - WT in auxin mutant group showing delayed germination**
